# A comparison of techniques for deriving clustering and switching scores from verbal fluency word lists

**DOI:** 10.3389/fpsyg.2022.743557

**Published:** 2022-09-14

**Authors:** Justin Bushnell, Diana Svaldi, Matthew R. Ayers, Sujuan Gao, Frederick Unverzagt, John Del Gaizo, Virginia G. Wadley, Richard Kennedy, Joaquín Goñi, David Glenn Clark

**Affiliations:** ^1^Department of Neurology, Indiana University, Indianapolis, IN, United States; ^2^Department of Psychiatry, Richard L. Roudebush VA Medical Center, Indianapolis, IN, United States; ^3^Department of Biostatistics, Indiana University, Indianapolis, IN, United States; ^4^Department of Psychology, Indiana University, Indianapolis, IN, United States; ^5^Biomedical Informatics Center, Medical University of South Carolina, Charleston, SC, United States; ^6^Department of Medicine, University of Alabama at Birmingham, Birmingham, AL, United States; ^7^Weldon School of Biomedical Engineering, Purdue University, West-Lafayette, IN, United States

**Keywords:** dementia – Alzheimer’s disease, verbal fluency, neuropsychology, language, Bayesian analysis

## Abstract

**Objective:**

To compare techniques for computing clustering and switching scores in terms of agreement, correlation, and empirical value as predictors of incident cognitive impairment (ICI).

**Methods:**

We transcribed animal and letter F fluency recordings on 640 cases of ICI and matched controls from a national epidemiological study, amending each transcription with word timings. We then calculated clustering and switching scores, as well as scores indexing speed of responses, using techniques described in the literature. We evaluated agreement among the techniques with Cohen’s κ and calculated correlations among the scores. After fitting a base model with raw scores, repetitions, and intrusions, we fit a series of Bayesian logistic regression models adding either clustering and switching scores or speed scores, comparing the models in terms of several metrics. We partitioned the ICI cases into acute and progressive cases and repeated the regression analysis for each group.

**Results:**

For animal fluency, we found that models with speed scores derived using the slope difference algorithm achieved the best values of the Watanabe–Akaike Information Criterion (WAIC), but with good net reclassification improvement (NRI) only for the progressive group (8.2%). For letter fluency, different models excelled for prediction of acute and progressive cases. For acute cases, NRI was best for speed scores derived from a network model (3.4%), while for progressive cases, the best model used clustering and switching scores derived from the same network model (5.1%). Combining variables from the best animal and letter F models led to marginal improvements in model fit and NRI only for the all-cases and acute-cases analyses.

**Conclusion:**

Speed scores improve a base model for predicting progressive cognitive impairment from animal fluency. Letter fluency scores may provide complementary information.

## Introduction

Among neuropsychological tests, verbal fluency tasks are widely recognized for their brevity, ease of administration, and diagnostic utility. The participant is asked to generate a list of words in response to a cue. The most common cues are semantic (e.g., “animals”) or an initial letter (e.g., “F”). The number of correct words produced within a time limit, usually 1 min, is termed the *raw score*. Raw scores measure the contributory cognitive processes as a single, informative value and are effective for detecting dementia (Canning et al., 2004) and for differentiating among potential causes of dementia (Tröster et al., 1989; Randolph et al., 1993; Monsch et al., 1994; Jones et al., 2006). Semantic fluency and letter fluency are often administered together for their contrast. Canning et al. (2004), for example, reported that simply generating more F-words than animals supported a non-vascular cause of mild cognitive impairment. In verbal fluency work comparing cognitively normal controls to patients with Alzheimer’s disease (AD), semantic dementia (SD), or another form of primary progressive aphasia (PPA), the contrast between letter and semantic fluency was helpful for differentiating among the three clinical syndromes. Semantic dementia patients patterned with PPA patients on semantic fluency (both significantly worse than AD), but with AD patients on letter fluency (both significantly better than PPA; Marczinski and Kertesz, 2006).

As with any cognitive test, performance on verbal fluency tasks likely depends on several dissociable cognitive skills, such as cognitive flexibility, psychomotor speed, semantic memory, and information retrieval (Pekkala et al., 2013; Eastman et al., 2014; Vonk et al., 2019). To measure these processes, investigators have developed new scoring methods that take into account qualities of the generated word lists. The two most widely recognized of these novel scores are predicated on the supposition that performance on verbal fluency relies on at least two dissociable processes: *clustering*, a relatively rapid, automatic transition between highly associated words (e.g., *dog* – *cat*), and *switching*, a slower, deliberate transition from a group of related words to an unrelated group (e.g., farm animals to ocean animals; Troyer et al., 1997; Troyer, 2000). Research on young, unimpaired subjects suggests that both of these scores depend on working memory capacity. However, clustering also depends on vocabulary, while switching depends on processing speed (Unsworth et al., 2011). These findings coincide with a previous observation that healthy older individuals exhibit lower switching scores than younger individuals, but have comparable clustering scores (Troyer et al., 1997), as one might expect based on other work indicating that normal aging impacts processing speed and executive function (Keys and White, 2000), rather than vocabulary (Hayden and Welsh-Bohmer, 2011; Harada et al., 2013). These cognitive observations fit well with the findings that individuals with frontal lobe lesions exhibit reduced switching scores, while those with temporal lobe lesions (especially on the left) exhibit reduced clustering scores (Troyer et al., 1998a).

The truth of the matter is likely more complex than suggested by these observations, and other investigators have proposed an alternative dichotomy consisting of semantic search and non-semantic factors (e.g., task difficulty, executive function; Mayr and Kliegl, 2000; Mayr, 2002). These investigators propose a regression model with the interword interval (IWI) as the dependent variable and the index of the interval as the independent variable. Thus, if an individual generates the list “dog – cat – lion,” there would be two IWIs, with indices 1 and 2, with the first occurring between the words dog and cat and the second occurring between the words cat and lion. Mayr and Kliegl (2000) propose that the slope coefficient for this model relates to semantic search, which grows increasingly difficult as the task continues, resulting in increasingly long IWI. The intercept term, however, corresponds to unchanging, non-semantic factors.

A key step in the calculation of clustering and switching scores is the identification of *linkages* between words. By linkage, we refer to a strong relationship in terms of meaning, sound, or spelling. Researchers have employed several techniques to accomplish this goal. The most common method uses human raters to identify linkages through direct examination of word lists (Troyer, 2000; Murphy et al., 2006). For example, words may be considered semantically linked because they refer to taxonomically (*lion/tiger*) or phenotypically (*shark/dolphin*) similar entities, entities seen in a similar context (*horse/cow*), predator–prey relationships (*cat/mouse*), etc. Phonological linkages consist of homonyms (*for/four*), rhymes (*freight/fate*), and words differing in only one vowel sound (*fit/fat*). Orthographic linkages consist of words beginning with the same two letters (*first/fidget*). Good inter-rater reliability has been reported with human raters (Abwender et al., 2001; Ross et al., 2007) but test–retest reliability (i.e., stability of scores in the same individual over repeated testing) may suffer due to learning effects (Ross et al., 2007). Some investigators have automated the identification of semantic linkages by first creating a structured list of subcategories and words belonging to each subcategory, then using a computer program to identify clusters and calculate scores (Clark et al., 2014, 2016). Identification of orthographic and phonological linkages is readily automated with functions on strings of letters or phoneme symbols from the ARPAbet (Jurafsky and Martin, 2009). This computerized method does not differ in principle from the original method of identifying clusters described by Troyer et al. (1997), but guarantees uniform determination of word linkages across participants. Thus, other investigators could readily reproduce this computerized method with no concern of poor inter-rater reliability.

A second technique involves the definition and application of some objective metric of similarity between words, such as semantic (Pakhomov et al., 2012) or phonological similarity (Ryan et al., 2013). One defines a pair of words to be related if their measured similarity exceeds a certain threshold. Semantic similarity is typically computed using methods developed for distributional semantics, in which one creates a vector representation for a word’s meaning based on other words that co-occur with the target word in a corpus of written text. The vectors may be derived by point-wise mutual information of neighboring words in a corpus (Clark et al., 2014), a holographic method (Jones and Mewhort, 2007), Latent Semantic Analysis (Pakhomov and Hemmy, 2014), or various methods related to artificial neural networks, such as word2vec (Mikolov et al., 2013) or global vectors (GloVe; Pennington et al., 2014). Similarity between two semantic vectors may then be measured by calculating the normalized dot product, which is identical to the cosine of the angle between the two vectors. Thus, identical vectors have an angle of 0°, for which the cosine is 1. Words with unrelated meanings are expected to have an angle near 90°, for which the cosine will be near 0. A network may then be induced on the set of target words by mapping each word to a vertex, measuring the similarity between each pair of words, and adding an edge between the two vertices if the similarity exceeds a threshold defined by the investigator. A series of consecutive words in a verbal fluency list may be considered a cluster if the corresponding vertices compose a complete subgraph of the network. Any transition away from the current complete subgraph would be considered a switch. Networks may be derived using other (i.e., non-semantic) objective criteria. In this work, we describe a method for deriving letter fluency networks by setting thresholds on three word-similarity metrics.

In a similar vein, Goñi et al. (2011) describe a network-generating method of three steps. In the first step, preliminary edges are established based on a probabilistic analysis of word proximities in a corpus of fluency word lists. The initial linkages are not limited to semantic associations and could represent any association commonly made by the participants, including sound or spelling relationships. In the second and third steps, the preliminary network is enriched with additional edges (see Methods for more details). Like the other network-generating methods described above, this technique does not rely on potentially idiosyncratic human judgments.

Another data-driven approach to identifying word linkages rests on the assumption that consecutive words separated by shorter intervals of time are related, while those separated by longer intervals are not. This method is implemented *via* the *slope difference algorithm* (Bousfield and Sedgewick, 1944; Gruenewald and Lockhead, 1980; Rosen et al., 2005), which seeks to account for each individual’s diminishing rate of word retrieval over the course of the task (Meyer et al., 2011; Lenio et al., 2016). This method has two potential disadvantages and two potential advantages. The first disadvantage is that this method requires the potentially labor-intensive, precise measurement of the latency for each word generated. The second disadvantage is that the word linkages vary from one individual to the next, or even from one trial to the next. The first advantage is that, like the Goñi method, the slope difference algorithm does not rest on potentially idiosyncratic human judgments. Second, there is no need to analyze a corpus of fluency word lists or other written material, as linkages are identified by timings alone.

Timing of verbal fluency responses has received limited attention in the analysis of verbal fluency for prognostic purposes. One approach to assessing the prognostic value of markers is to compare individuals with increased genetic risk for dementia. Such a comparison of apolipoprotein E ε4 carriers to non-carriers reveals that carriers produce smaller clusters of words than non-carriers, and take longer to access those clusters (Rosen et al., 2005). Recent work suggests that scores calculated from verbal fluency word timings are valuable for differentiating MCI patients from individuals with normal cognition (König et al., 2015; Chen et al., 2020). Similarly, “pause length” (ignoring intervals shorter than 250 ms) contributes to machine learning regression models predicting scores on the Mini-Mental State Exam (Folstein and Folstein, 1975) and Clinical Dementia Rating (Morris, 1993) sum of boxes (Linz et al., 2017). In other work on the same data set analyzed here, we observed that the speed of word generation during verbal fluency improves estimates of time to ICI (Ayers et al., 2022). However, the speed scores we investigated previously incorporated *all* word generation times rather than separating transitions into those between linked words (which we term “edge” transitions) and those between unlinked words (“switch” transitions), as we do in these analyses. The approach of Mayr and Kliegl (2000) was developed for theoretical considerations and the study of normal aging, but here, we explore its utility for prognostic purposes.

Data for this analysis come from the Reasons for Geographic and Racial Differences in Stroke (REGARDS) study (Wadley et al., 2011; Howard, 2013), a longitudinal epidemiological study following 30,239 volunteers, with approximately equal recruitment of white and African-American individuals (Howard et al., 2005). We identified a subset of participants with ICI and matched them to controls on demographic parameters. We then transcribed telephone-based verbal fluency recordings from each case and control. Acquisition of linguistic data by telephone raises the possibility of developing approaches to remote assessment for early detection of dementia, perhaps even before the onset of symptoms. Such approaches could be used to inexpensively screen individuals for treatment, clinical trials, or observational studies. Fluency tasks administered by telephone correlate well with those administered in person (Bunker et al., 2017).

In this work, we compare three techniques for identifying linkages between words generated during animal and letter F fluency tasks: those originated by Troyer, network-based techniques, and slope difference. In addition, for each technique, we evaluate scores based on the speeds of edge and switch transitions. To these timing-based scores, we add scores calculated using the technique described by Mayr and Kliegl (2000). First, we evaluate the amount of agreement among the techniques in terms of the linkages they define. Second, we calculate clustering, switching, and speed scores based on the defined linkages and examine the correlations. Third, to assess the empirical value of scores calculated with the various techniques, we compare Bayesian logistic regression models fit with each set of scores, with the outcome of ICI. Such a model, if highly accurate, could contribute meaningfully to approaches for dementia risk stratification. We compare the resulting models on an array of quality metrics. After identifying the best model for the category and letter fluency tasks, we fit a final model with the union of the two sets of variables and examine the posterior distribution. Finally, to account for the presumed heterogeneity of our sample, we partition the cases into those that exhibit acute decline and those that exhibit progressive decline, repeating the analysis for each subset of cases.

## Materials and methods

### The REGARDS study and participants

We identified 640 cases of ICI (defined below) among participants in the REGARDS study (Howard et al., 2005). We considered only subjects who were clinically stroke-free and who had completed both animal and letter fluency assessments. Incident cognitive impairment was determined by the pattern of longitudinal scores on the Six-Item Screener (SIS). The SIS is a brief test in which the participant is given three words to remember, then undergoes testing with three temporal orientation items, followed by recall for the three words. The three temporal orientation items and three recall items are worth 1 point each (six points max). We defined an abnormal score on the SIS to be less than five points (sensitivity for cognitive impairment 74.2% and specificity 80.2%; Callahan et al., 2002). We identified suitable cases of ICI according to two criteria: (1) normal SIS scores up until at least one assessment dated *after* the first complete verbal fluency evaluation and (2) at least one subsequent abnormal SIS score, including an abnormal score at the most recent SIS evaluation. Criterion 1 was selected to ensure that individuals were cognitively normal at the time of the verbal fluency assessment. Criterion 2 was selected because it was used in previous work on ICI in REGARDS (Wadley et al., 2011) and because it guards against including individuals who do not exhibit consistent impairment in cognition.

We matched ICI cases with controls from a pool of 14,281 stroke-free individuals who never received an abnormal SIS score during the years they were evaluated. Matching took place based on age, sex, education, race, and geographic region. We matched cases to controls with an absolute age difference <3 years. Educational level was categorized coarsely as “less than high school,” “high school graduate,” “some college,” or “college graduate or more.” The REGARDS study divides participants according to whether they reside in one of three geographic regions that relate to an individual’s stroke risk. The key defining region is the “stroke belt,” which includes most of the southeastern states, as well as Indiana, in which individuals suffer from increased stroke risk. Stroke risk is even higher in the second region, composed of the coastal plains of North Carolina, South Carolina, and Georgia, known as the “stroke buckle.” Individuals residing outside of either of these regions are assigned the regional category “nonbelt.”

**Figure 1:**
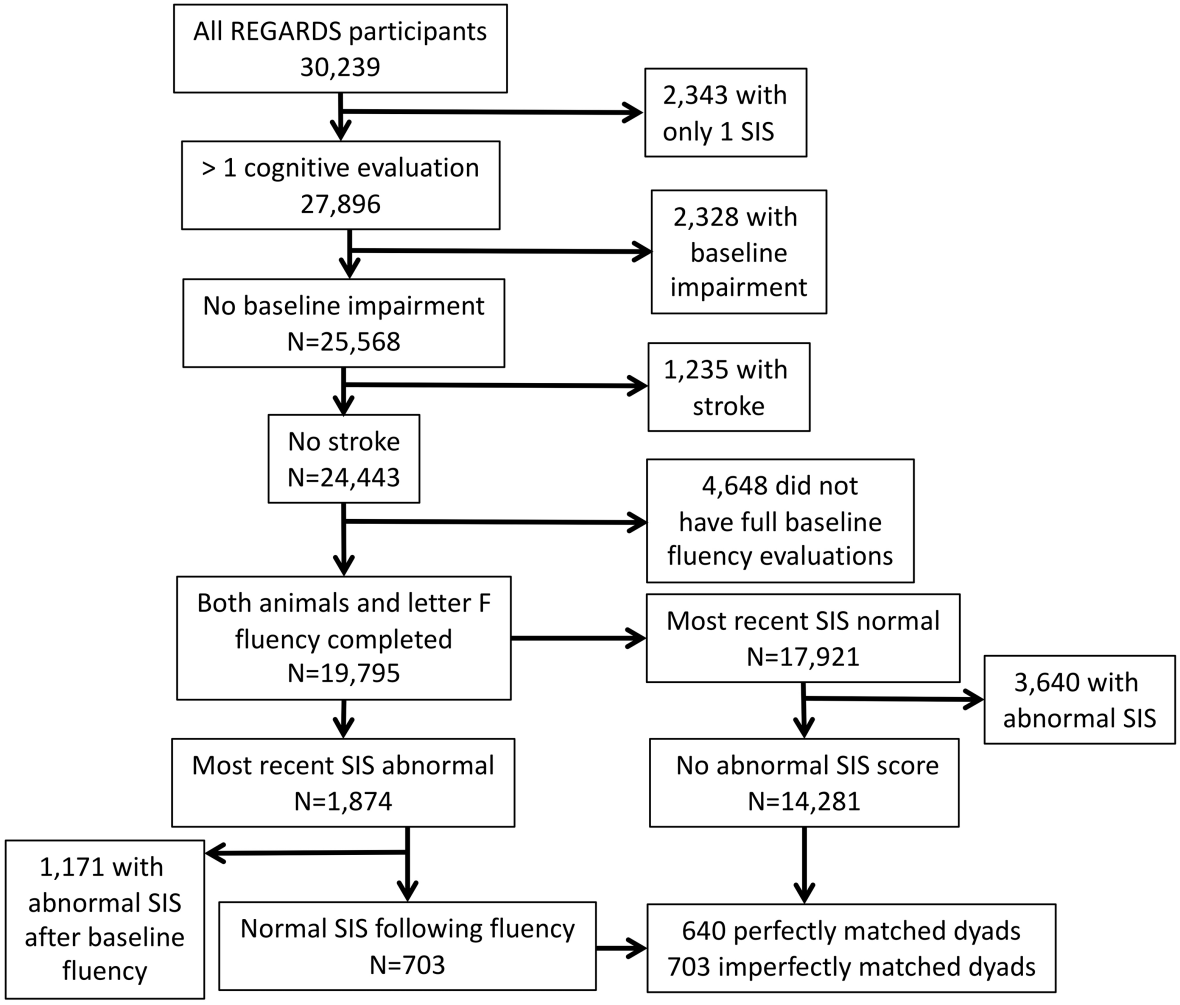
Flow chart depicting participant selection. Individuals were considered for the case group only if SIS scores were normal prior to recording the fluency tasks *and* at the first SIS follow-up after the fluency recording. This stipulation ensures that SIS performance was normal at the time of the fluency recording.

Using the R library BayesFactor (Morey, 2018) we assessed quality of the match by calculating Bayes factors on the demographic variables. We took the logarithm of the Bayes factors to make them symmetric and to constrain the magnitude of our reported values. With traditional Bayes factors, a value of 10 indicates that the alternative hypothesis is 10 times more likely than the null, a value of 0.1 indicates that the null hypothesis is 10 times more likely than the alternative, and 1 suggests a paucity of evidence in either direction. After taking the base 10 logarithm, these values become 1, −1, and 0, respectively. We anticipated evidence of no effect of group membership (log BF < −1) on each demographic variable (Keysers et al., 2020). We were able to perfectly match 640 of the ICI cases to controls, yielding a final sample of 1,280 participants (see Figure 1). We incorporated demographic variables into all regression models as covariates.

The REGARDS Study was approved by the Institutional Review Board of the University of Alabama at Birmingham and this analysis was approved by the Institutional Review Board of Indiana University.

### Data pre-processing

We undertook a sequence of steps to derive an accurate transcription of each verbal fluency recording. First, we obtained a preliminary transcription with time stamps of all 2,560 REGARDS audio files using the Amazon Web Services Transcribe tool. These transcriptions were about 61% accurate and occasionally omitted clearly audible utterances. Second, we applied a voice activity detection tool to refine the timings and identify omitted material. Third, human raters checked and corrected the preliminary transcriptions using a custom MATLAB-based (Natick, MA) graphical transcription tool, labeling utterances made by the test administrator to ensure that they could be analyzed separately from those of the study participant. Raters were blinded to clinical outcome and received recordings in batches with random mixtures of cases and controls. Ambiguities and poorly understood utterances were resolved informally by a consensus of raters and study investigators.

### Scoring methods

#### Raw, repetition, and intrusion scores

Repetitions and intrusions were identified manually during the transcription process. Each item was assigned a canonical form corresponding to the unabbreviated and uninflected form of the word. Utterances with the same canonical form as a previous utterance in the list were labeled as repetitions. For animals, words that could not be interpreted as a type of animal were regarded as intrusions. For F-words, we regarded as intrusions proper nouns (e.g., *France*) and words not starting with the letter F (e.g., *phone*). These errors must be identified during calculation of the raw score, and are likely to provide additional information with diagnostic relevance (Suhr and Jones, 1998; Wajman and Cecchini, 2022). Thus, we wished to perform our evaluations of model quality while accounting for these scores, because they are easy to calculate and the work necessary to tabulate them is obligatory when deriving an accurate raw score. Raw, repetition, and intrusion scores were extracted automatically from the transcription file.

#### Clustering and switching methods

##### Troyer methods

We employed automatic methods to calculate clustering and switching scores (Clark et al., 2014, 2016). Automation of the process did not qualitatively alter any judgments of word relatedness, but ensured that these judgments were applied uniformly across the entire sample. For animal fluency, this method required the *a priori* definitions of animal subcategories starting from subcategories given by Troyer (2000). Animals were included as members of multiple subcategories when appropriate (e.g., beaver may be classified in subcategories *rodent*, *water creature*, and *used for fur*). A cluster is defined as a sequence of words belonging to a common subcategory.^1^ (See Supplementary Table S1 for the list of subcategories and animals assigned to each subcategory).

For letter fluency, the method required automatic identification of pairs of words meeting any of four criteria: those that rhyme, share the same first two letters, are homonyms, or differ only by a vowel sound (Troyer et al., 1997; Troyer, 2000; Murphy et al., 2006). To this end, we defined predicates to automatically make these determinations by acting on the word strings or on the pronunciations from the CMU pronunciation dictionary (CMU, 2014). An electronic archive file containing a complete list of F-words generated by participants in this study, along with their ARPAbet pronunciations is available by request from the corresponding author.

##### Network methods

Troyer’s original method identifies semantic relationships in the context of semantic (category) fluency and phonological or orthographic relationships in the context of letter fluency. However, semantic factors are likely the strongest influence on both category and letter fluency (Clark et al., 2014) and other forms of lexical similarity may influence either task in a clinically meaningful way (Abwender et al., 2001; Clark et al., 2016). Our second approach to calculating clustering and switching scores entailed the data-driven construction of network models describing word relationships, while leaving open the nature of these relationships. While the end products for both the semantic and letter fluency tasks were networks with unweighted and undirected edges, we employed different methods to construct them.

For animal fluency, we followed the method described by Goñi et al. (2011), first identifying which pairs of words in our corpus of verbal fluency word lists occurred in proximity to one another more often than predicted by chance alone. Of note, this step may identify any form of word relationship (e.g., semantic, phonological, or even those defined by alphabetical ordering, i.e., *aardvark*, *bat*, *cat*, *dog*…). We used a window length of two and an alpha level of 0.001 to derive a preliminary network. This preliminary network excluded most animal word types in our corpus, as they had not occurred enough times to overcome any reasonable probability threshold. Following Goñi, we took three additional steps to enrich and expand this network. We first employed a generalized topological overlap measure (GTOM). This algorithm established new edges between vertices that were distanced no more than *m* words apart (where *m* is the order of GTOM) in the preliminary network. From these added edges, an overlap measure was computed, and the resulting matrix was transformed into a dissimilarity matrix. We then identified modules in the dissimilarity matrix by applying hierarchical clustering and enriched the newly defined modules by adding edges between all pairs of words within each module. Finally, in a step not taken in Goñi’s original work, we further expanded the network by placing all word types excluded from the original network into one of the modules. To do so, we first matched each word in the corpus with its corresponding “global vector” (GloVe). These vectors are 300-dimensional numerical representations of word meaning derived by training a neural network on word co-occurrences in a large corpus of English (Pennington et al., 2014). We then calculated the cosine similarity between the GloVe of the new word and all words in the network, placed the new word into the module containing its closest semantic neighbor, and then fully integrated that word into the module by adding edges to all other words in the module.

Letter fluency depends less heavily on semantic associations than animal fluency and relatively more heavily on phonological or orthographic similarities, although the semantic influence remains the strongest (Clark et al., 2014). In addition, we anticipated that networks reflecting mental associations for these two tasks would differ in terms of overall network size, node degree, clustering tendency, and average path length. Empirically, we were skeptical that enrichment with techniques such as GTOM and HCA would give coherent results for letter fluency. For example, if the initial probabilistic step were to produce edges establishing the path *frigate – freight – fate*, GTOM2 would introduce a dubious edge between *frigate* and *fate*. Preliminary attempts to apply the Goñi method to letter fluency data yielded a network with questionable validity, as it included only 66 of the 867-words generated.

As an alternative, we constructed a network of F-words in which it was possible for edges to represent any of three possible lexical relationships: semantic, phonological, or orthographic. We began with three separate letter F networks, each of which had a vertex for each word in our dataset. Each edge was weighted with the similarity between the two words at the vertices connected by the edge. For the semantic network, we defined semantic similarity as the cosine similarities of the GloVes corresponding to each pair of words. For the orthographic network, we calculated the Levenshtein edit distance (Levenshtein, 1966) and multiplied it by −1 to obtain a similarity measurement. To quantify phonological similarity, we employed the Needleman–Wunsch algorithm (Needleman and Wunsch, 1970), a dynamic programming algorithm similar to the Levenshtein edit distance, but incorporating information about the similarity of sequence elements when determining the optimal alignment (e.g., *G* is more similar to *K* than it is to *SH*). For details of this algorithm, see the Python code in the Supplementary File. Working code and necessary data files are available by request.

From our three weighted networks, we wished to derive a single, unweighted network defining linkages among all listed F-words. To do so, we needed to set a threshold for each form of similarity. The final network would then be defined as having an edge between any pair of vertices (words) for which some form of lexical similarity was above the threshold. We set the goal of identifying a threshold for each similarity metric that would maximize the agreement between the final network and human raters on a subset of the data. Two authors (JB and DGC) provided subjective binary judgments of relatedness for 484 pairs of F-words that occurred in 50 randomly selected verbal fluency word lists (25 NC, 25 ICI). We placed the three networks on a uniform scale by z-transforming the weights and performed a grid search over possible semantic, orthographic, and phonological thresholds (using a step size of 0.05 standard deviations), seeking to maximize the average kappa between the resulting adjacency matrix and the human raters.

For both animal and letter fluency, clustering and switching scores were calculated from each graph by considering any consecutive sequence of words to be a cluster if the vertices corresponding to those words composed a fully connected subgraph. Cluster size was defined as one less than the number of words in such a sequence. According to this definition, a word that has no edge connecting it either to the preceding or subsequent words in the list is considered to be a complete graph of one vertex, and thus is a cluster of size 0. We defined the number of switches to be one less than the number of clusters.

##### Slope difference

The slope difference algorithm is a data-driven approach to identifying related items that relies on measurement of timings between words. Following Bousfield and Sedgewick (1944) and Gruenewald and Lockhead (1980), we first expressed each individual’s performance as a function of increasing raw score over time. We then used a MATLAB program to fit an exponential curve to this function by determining values of *c* and *m* that minimized the sum of squared differences between the raw score curve and the formula *y* = *c*(1 – *e^–mt^*), where *t* represents the latencies of the word onsets and *e* is the base of the natural logarithm.

**Figure 2:**
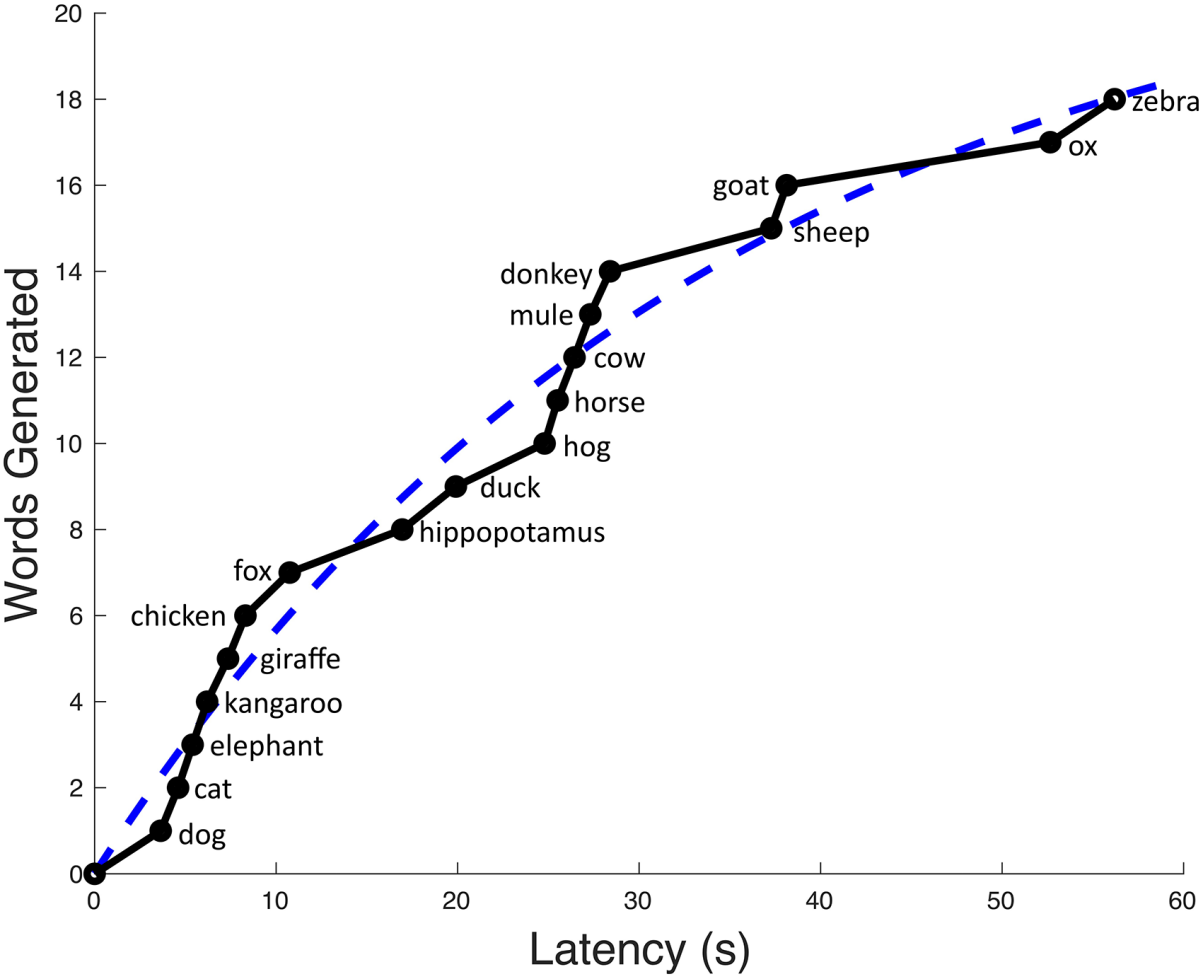
The black, jagged curve depicts the number of animal words actually generated over time. To this curve, we fit an exponential curve (blue dashes). The chains of animals linked based on positive slope difference are: [dog, cat, elephant, kangaroo, and chicken], [fox], [hippopotamus], [duck], [hog, horse, cow, mule, and donkey], [sheep and goat], [ox and zebra]. This list yields a switching score of 6 and a mean chain size of 1.43.

This curve served as the prediction of expected output over time (see Figure 2). The program then compared the slopes of these two curves at time points halfway between each consecutive pair of words. Words that were produced faster than predicted by the exponential curve (positive slope difference) were considered to be linked to the previous word. Those produced more slowly than predicted (negative slope difference) were considered to be switches. “Clusters” defined by this method are based only on connections between consecutive words, as there is no clear way to extend the method to determine whether non-consecutive words should be linked. When specifically discussing clusters derived from slope difference, we will use the term “chains.”

##### Switch-edge speeds

The Troyer, network-derived, and slope difference techniques were used for establishing which words were linked, and the sets of linkages were used to calculate clustering and switching scores for each technique. We wished to extend these methods to derive new, timing-based scores. To this end, we computed speed scores by measuring the time elapsed during edge transitions (when consecutive words are linked) and during switch transitions (when consecutive words are not linked).

The distribution of IWI was positively skewed and was normalized by taking the fourth root. We partitioned all transitions into those between linked words and those between non-linked words. We then calculated speed scores from each of these two sets of transformed intervals by first performing min-max normalization (which forces all values onto the interval [0, 1]), then inverting the normalized intervals by subtracting them from 1.0. Thus, the fastest transition received a score of 1.0 and the slowest transition received a score of 0.0. We then derived two summary speed scores by summing the inverted, normalized switch and edge speeds for each participant.

##### Mayr scores

Following Mayr (2002) and Mayr and Kliegl (2000), we computed a simple linear regression for each individual word list with IWI as the dependent variable and the index of the IWI (i.e., first IWI index = 1, second IWI index = 2, etc.) as the independent variable. The slope term was recorded as the individual’s semantic search score (S) and the intercept was recorded as a measure of constant, non-semantic factors affecting the individual’s speed on the task (C). We measured IWI only between consecutive pairs of valid words.

### Agreement and correlation among scoring methods

We calculated Cohen’s κ between each pair of techniques, for each fluency task. For the Troyer method, words listed together in any subcategory received a “1.” For the network methods, every pair of words in the edge set of the graph received a “1.” Because the slope difference algorithm variably assigns linkages, and therefore a given pair of words may be linked in some lists and not in others, a ‘1’ was assigned to any pair of words that were determined to be linked at least half of the times they occurred consecutively. Pairs of words that were not linked were assigned a ‘0.’

We generated correlation matrices to compare clustering, switching, and speed scores across all scoring methods. As all these scores are likely to correlate with raw score, we used regression to remove the influence of raw score from each clustering, switching, or timing score prior to calculating the correlations.

### Empirical ranking of scoring methods

Apart from straightforward comparison of methods for calculating clustering and switching scores, we wished to ascertain whether any method had superior empirical value. There are many potential uses for clustering and switching scores, but we focused specifically on the question of identifying individuals at risk for cognitive impairment. Toward this end, we fit a Bayesian logistic regression on each of the candidate models using a quadratic approximation in the rethinking library for R (McElreath, 2020). We chose this statistical approach for four reasons. First, logistic regression is very familiar to most researchers in neurology and psychology and is straightforward to interpret. Second, the Bayesian approach, while giving results very comparable to traditional logistic regression when sample sizes are sufficiently large, yields a probability distribution for each coefficient. These probability distributions have a more intuitive interpretation than traditional confidence intervals. Third, the posterior probability distributions emitted by these models may serve as prior distributions for future analyses. Fourth, the Watanabe–Akaike Information Criterion (WAIC; Watanabe, 2010) is quick to compute and provides a simple metric for model comparison. As an estimate of the out-of-sample deviance, the WAIC in practice yields results similar to those obtained with leave-one-out cross-validation (McElreath, 2020). Given a set of models with WAIC measures, a weight may be computed for each model that corresponds to the probability that the model will minimize the information loss relative to the other models.

The outcome variable for all regression models was ICI, as defined in the section above detailing cognitive data obtained from the REGARDS study. We began with a base model for each verbal fluency task (animals and letter F), consisting only of demographic variables, raw score, repetitions, and intrusions. All coefficients were assigned an uninformative, normal prior with zero mean and standard deviation of 100. We computed three additional models by adding clustering and switching scores to each base model, based on a Troyer technique, a network technique, or the slope difference technique. We then computed the four models incorporating timing-based scores. Thus, we studied a total of eight regression models for each fluency task. As noted for the correlation analysis, we redefined each novel score as the residual after regressing the novel score on raw score. This step ensured that the resulting coefficients were not inflated due to correlation with raw score and facilitated their interpretation.

We assessed the performance of each regression model with WAIC, relative model weight, area under the receiver operating characteristic curve (AUC), sensitivity, specificity, positive predictive value, negative predictive value, F1 score, and net reclassification improvement (NRI; Pencina et al., 2008). We repeated these analyses with only the acutely declining cases and again with only the progressively declining cases, maintaining the entire sample of controls across analyses for uniformity of comparison.

We selected the best performing animal and letter fluency models to combine into a single model and obtained precise posterior distributions for the regression coefficients with Markov chain Monte Carlo, employing freely available R software accompanying a textbook of Bayesian statistics (Kruschke, 2015). This software provided some important conveniences, including diagnostic plots for the Markov chains and automatic calculation of important metrics such as effective sample size and 97% highest density interval (HDI) for each coefficient. We illustrated the posterior and 97% HDI with a histogram for each regression coefficient.

## Results

### Subject group comparisons

Table 1 shows demographic and traditional scores on our participant sample. We took the base 10 logarithm of the Bayes factor (log BF) for each comparison. Negative values represent evidence in favor of the null hypothesis (i.e., that there is no difference between groups) and positive values represent evidence against the null hypothesis. A log BF of 0 indicates no evidence for or against the null hypothesis. Absolute values >0.48, 1, and 2 moderate, strong, and extreme evidence, respectively. When comparing the full sample of cases and controls, the evidence favored the null for all demographic variables. This pattern persisted when comparing acute decliners to controls. However, there was weak evidence of a greater proportion of females in the progressive decliner group and moderate evidence that progressive decliners were older than the controls.

**Table 1:** REGARDS participant data.

	Controls (*n* = 640)	All ICI (*n* = 640)	log BF	Acute (*n* = 536)	log BF	Progressive (*n* = 104)	log BF
Age (years)	74.96 (8.61)	74.99 (8.70)	−1.22	74.52	−1.01	77.52^*^	0.85
Sex (M:F)	293:347	293:347	−1.15	285:303	−0.95	39:76^*^	0.31
Region (Non-belt:Belt:Buckle)	258:242:140	258:242:140	−2.15	246:214:128	−2.06	44:45:26	−1.60
Education (<HS:HS:SC:CG+)	74:191:171:204	74:191:171:204	−3.00	69:179:160:180	−2.96	17:27:34:37	−1.72
Race (W:B)	397:243	397:243	−0.99	328:208	−1.13	69:35	−0.75
Animal fluency (words)	15.71 (5.16)	13.94 (4.94)^*^	7.06	14.13 (5.05)^*^	4.67	12.96 (4.16)^*^	4.56
Animal repetitions (words)	0.93 (1.38)	1.19 (1.71)^*^	0.69	1.15 (1.69)^*^	0.20	1.34 (1.82)^*^	0.62
Animal intrusions (words)	0.04 (0.22)	0.07 (0.37)	−0.68	0.07 (0.39)	−0.64	0.06 (0.27)	−0.85
Letter F fluency (words)	10.60 (4.21)	9.59 (4.25)^*^	2.63	9.59 (4.25)^*^	2.37	9.63 (4.29)^*^	0.03
Letter F repetitions (words)	1.03 (1.27)	1.17 (1.50)	−0.42	1.10 (1.40)	−0.96	1.52 (1.92)^*^	1.56
Letter F intrusions (words)	0.34 (0.69)	0.39 (0.84)	−0.84	0.40 (0.87)	−0.76	0.36 (0.67)	−0.92
Time to conversion or censoring (days)	1080.42 (451.12)	1001.38 (468.40)^*^	0.82	1023.79 (481.63)^*^	−0.26	885.88 (373.35)^*^	2.67
Number of SIS assessments	8.86 (2.01)	8.82 (2.03)	0.067	8.70 (2.10)	−0.79	9.25 (1.62)^*^	−0.19
Minimum SIS score	5.18 (0.38)	3.27 (1.12)^*^	229.3	3.54 (0.91)^*^	44.43	1.84 (1.06)^*^	38.82

All comparisons are between controls and one of the groupings of ICI (all ICI, acute, or progressive) participants. Metric variables are shown as mean (standard deviation) and compared with *t*-test. Categorical variables were compared using *χ*^2^. SIS, Six-Item Screener; ICI, incident cognitive impairment; log BF, base 10 logarithm of the Bayes factor; HS, High school education; SC, some college; CG+, college graduate and above. Base 10 logarithm of Bayes factor positive values are evidence against and negative values are for the null hypothesis. Absolute values 0–0.48 anecdotal, 0.48–1.0 moderate, 1.0–2.0 strong, >2.0 extreme evidence.

* *p* < 0.05 compared to controls.

Controls produced more animal words and more F words than cases, and cases repeated animal words more often than controls. Evidence that progressive cases produced fewer F-words than controls was much weaker (log BF 0.03) than for the acute cases or the full set of cases (both log BFs > 2). The opposite pattern held for letter F repetitions, where the evidence was that progressive cases produced more repetitions (log BF 1.56) than either of the other sets of cases (both log BFs negative). Cases and controls underwent comparable numbers of SIS evaluations, and therefore had to have been followed about the same length of time. However, those observed to have progressive decline had been followed longer than the controls (*p* < 0.05), although the log BF weakly favored the null hypothesis.

### Network models

**Figure 3:**
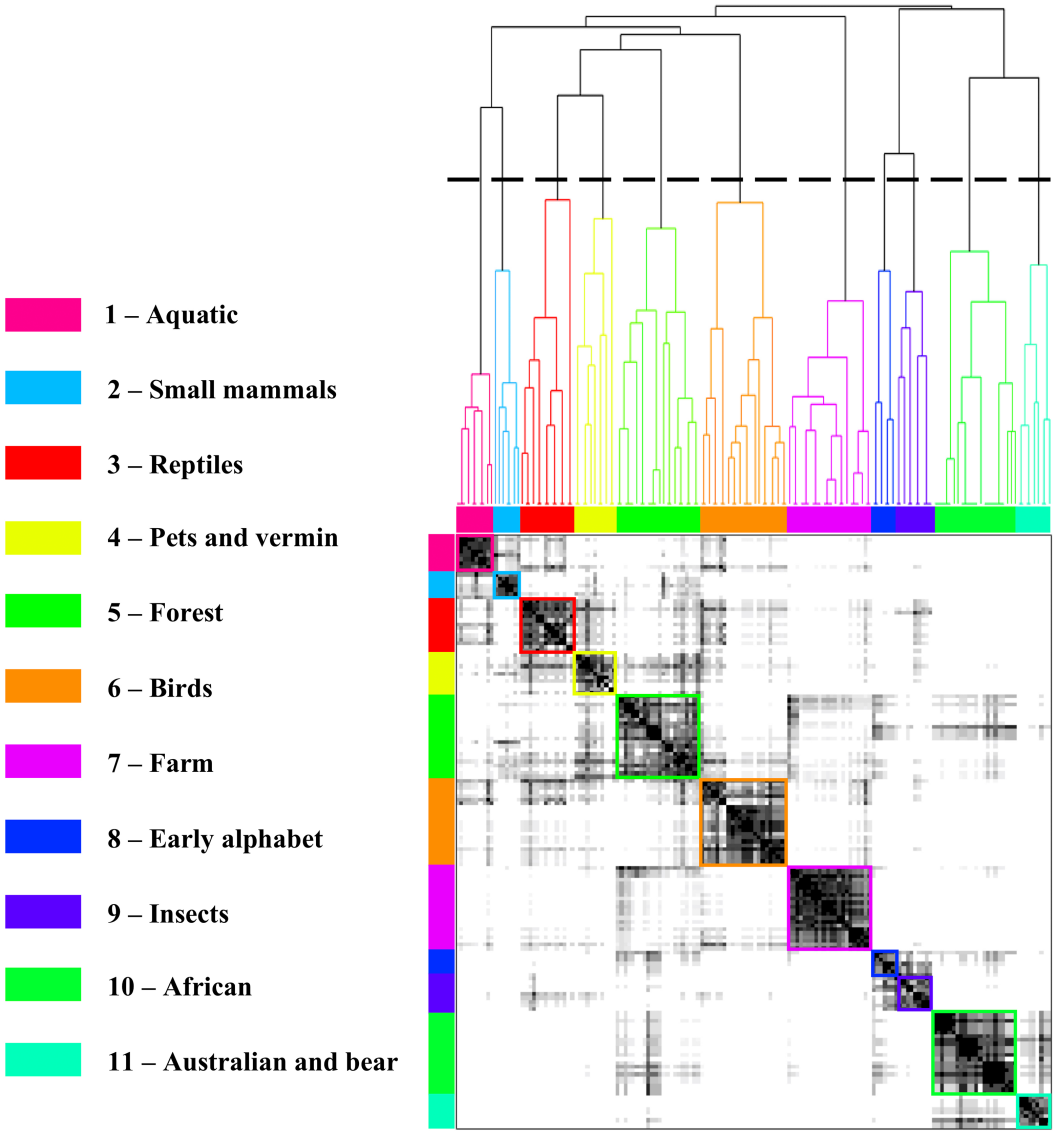
Adjacency matrix and dendrogram yielding 11 animal modules.

**Figure 4:**
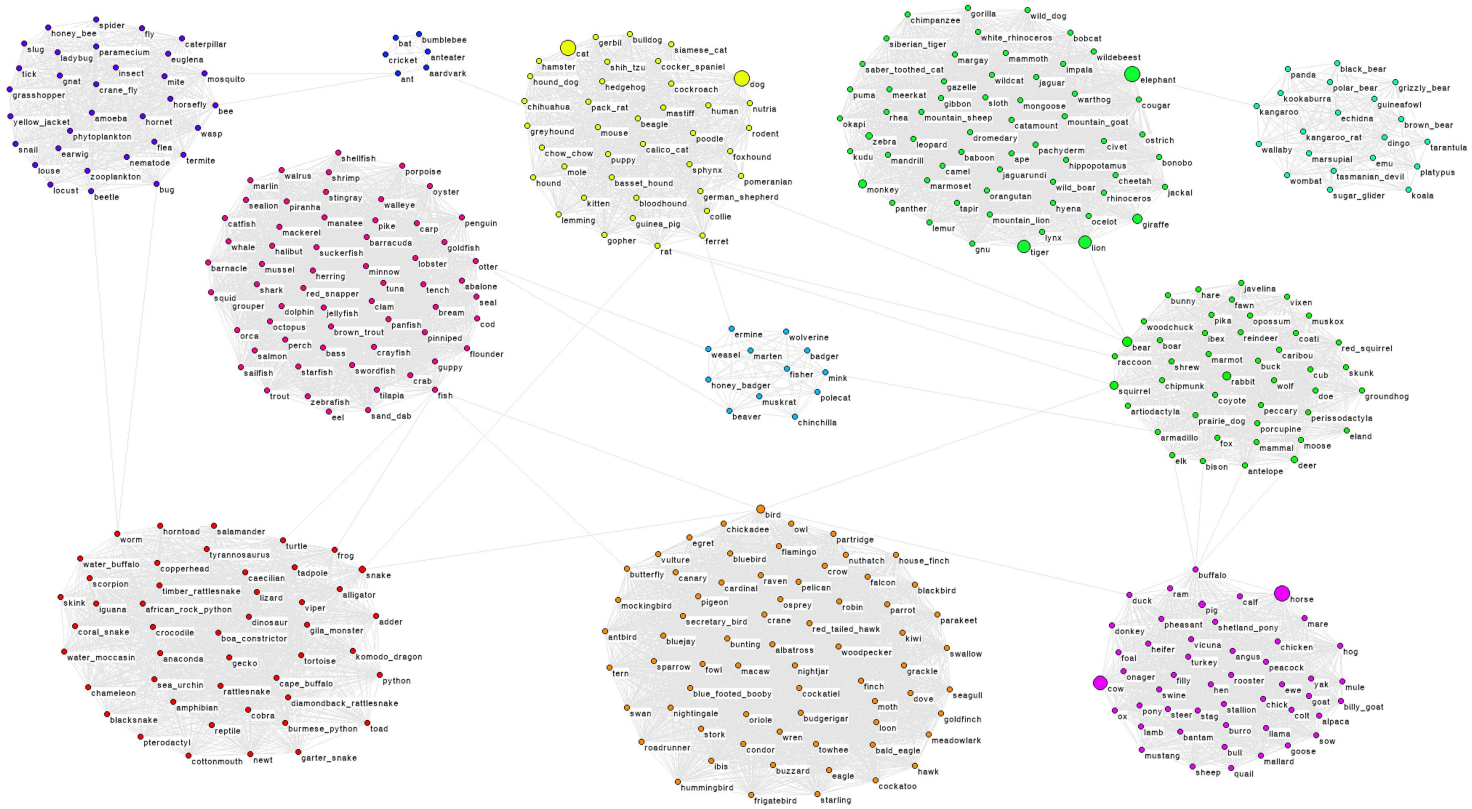
Final animal network. Animal names that were not included in the initial graph were placed in the module with their nearest semantic neighbor.

The total number of unique animals produced by all subjects was 433. The preliminary network was established using window length 2, *α* = 0.001, and GTOM2, and included 156/433 animals. We next applied hierarchical clustering, which yielded 11 modules (Figure 3). We added each of the remaining 277 words to whichever module contained the word’s closest semantic neighbor. Figure 4 shows the representation of the derived animal graph.

**Figure 5:**
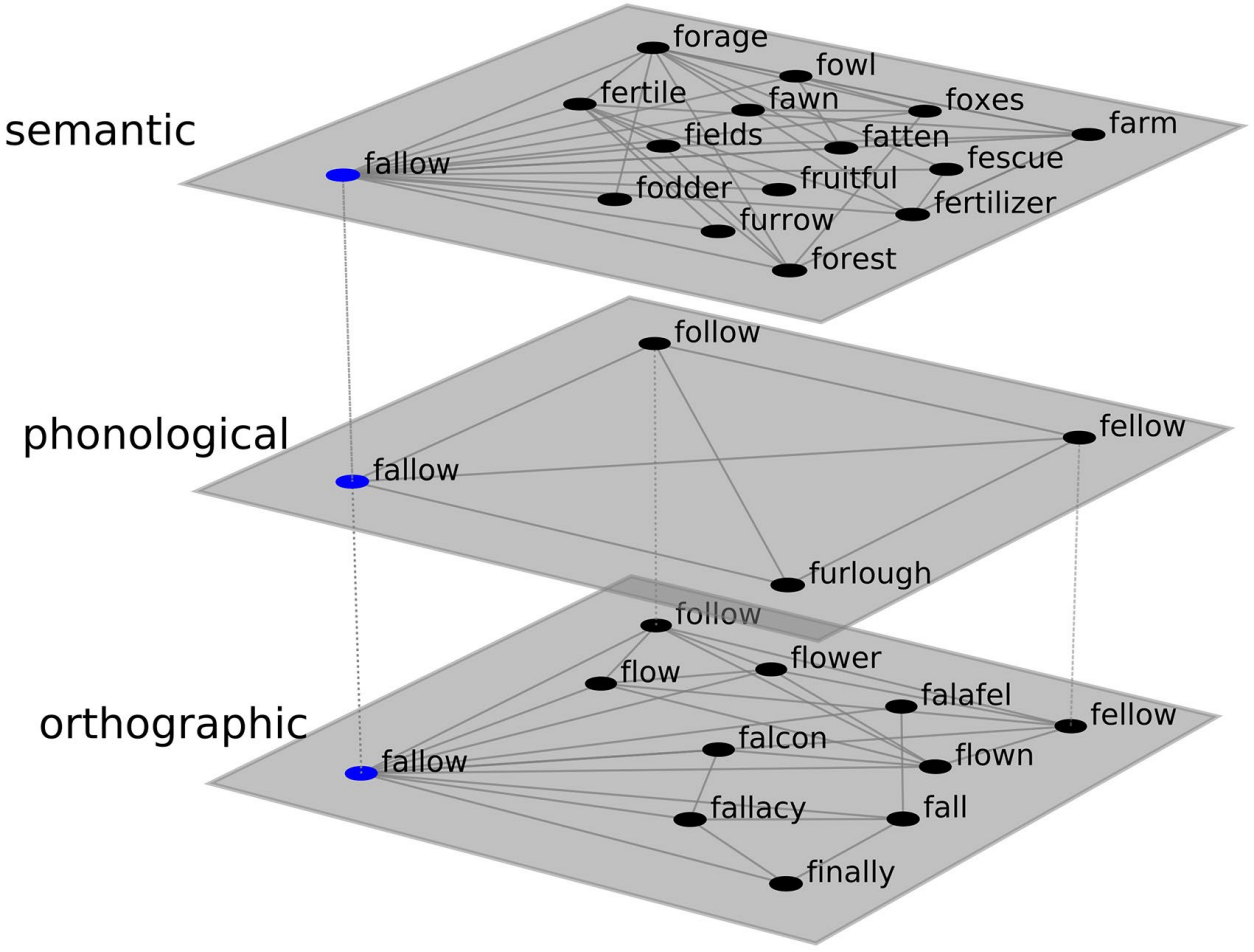
Representative portion of the letter F network, depicting semantic, orthographic, and phonological layers. Prior to calculating clustering and switching scores, these layers were collapsed into a single network by taking the union of edges.

The total number of unique letter F words (including morphological variations) produced by all subjects was 867. The κ score between the raters on 484 pairs of F-words was 0.60. The grid search to find z-thresholds for maximizing kappa between the network and human raters yielded a maximum average kappa score of 0.53 (DGC 0.55, JB 0.51) with a semantic threshold of +3.15, a phonological threshold of +1.70, and an orthographic threshold set at +2.00. See Figure 5 for a partial representation of the letter fluency graph.

The letter F graph was substantially larger than the animal graph in terms of both vertices (867 *vs*. 433) and edges (22,980 *vs*. 10,352). Average degree for F-words was somewhat higher than for animals (53.01 *vs*. 47.82). Animal words were more clustered than F-words (0.99 *vs*. 0.26), and the average path length was longer (4.02 *vs*. 2.19).

### Agreement and correlations

Agreement on relatedness of listed animals, as measured by Cohen’s κ, was fair between slope difference and the other two methods (~0.3), and was substantial between the Troyer and network-based methods (0.78). Agreement on relatedness of F-words was slight between slope difference and the other two methods (~0.05), but fair between the Troyer and network methods (0.34; see Table 2).

**Table 2:** Edge agreement of three methods.

	Graph	Troyer	Slope difference
Graph	–	0.78	0.30
Troyer	0.34	–	0.29
Slope difference	0.06	0.05	–

Kappa values in upper triangle are for animal fluency; those in lower triangle are for letter fluency.

**Figure 6:**
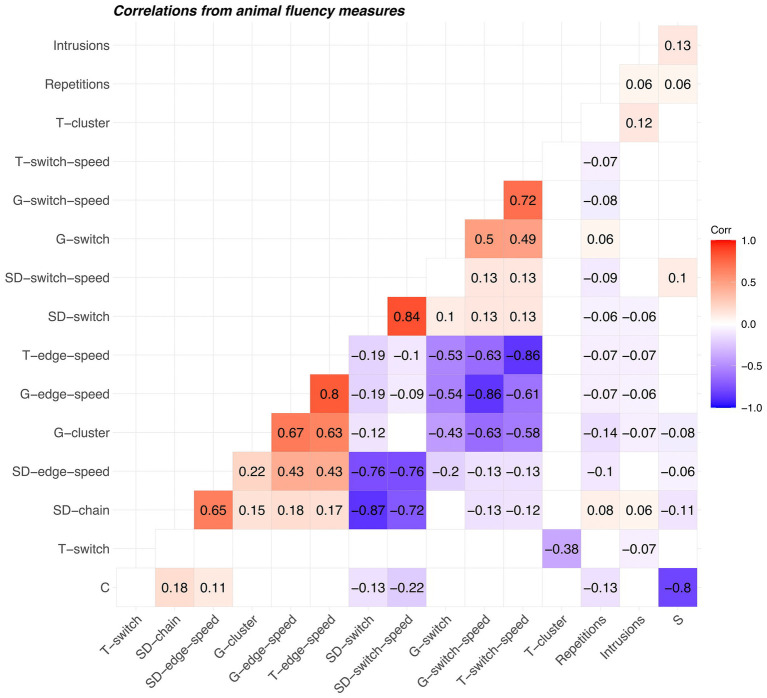
Correlation matrix of scores derived from animal fluency. Prior to calculating the correlations, the raw scores were regressed out of all scores tabulated here. T, Troyer method; G, graph-theoretic (network) method; SD, slope difference algorithm; C, Mayr constant term; S, Mayr slope term. SD “chain” scores are comparable to cluster scores, but are based only on linkages between consecutive words.

**Figure 7:**
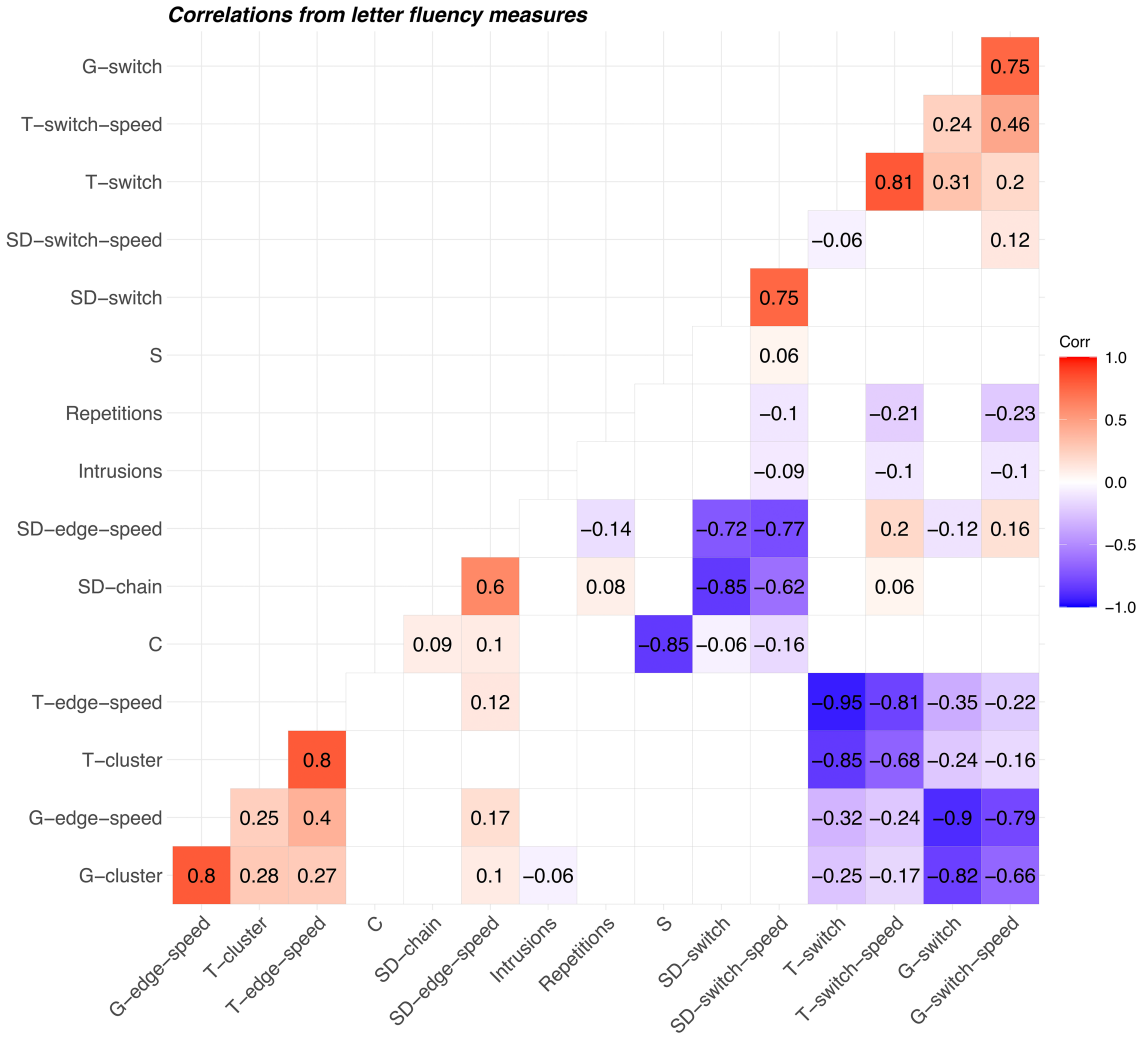
Correlation matrix of scores derived from letter fluency. Prior to calculating the correlations, the raw scores were regressed out of all scores tabulated here. Abbreviations are identical to those in Figure 6.

We calculated correlations after removing variance associated with raw scores (see Figure 6). Thus, if raw score were included in the matrices, it would have a correlation of 0 with all scores listed. Correlations between several pairs of corresponding scores derived with different techniques (Troyer and network-based) were high for animal fluency (*r* > 0.72). The strongest negative correlations for scores derived from animal fluency were between complementary scores derived using the same technique. For example, slope difference mean chain length (SD-chain) and slope difference switching (SD-switch) was −0.87. Similarly, the constant (C) and slope (S) Mayr scores showed a strong negative correlation (*r* = −0.80). For letter fluency, we found the strongest positive correlations between similar scores derived following the same method for identifying relatedness (see Figure 7); for example, between Troyer switching and Troyer switching speed (*r* = 0.81), between network-based clustering and network-based edge transition speed (*r* = 0.80). The strongest negative correlations also occurred within techniques, for example, between network-based edge transition speed and network-based switching (*r* = −0.90), between Troyer-style edge transition speed and Troyer-style switching (*r* = −0.95), and between the Mayr C and S scores (*r* = −0.85). Scores in both correlation matrices were arranged such that variables with similar patterns of correlation were placed adjacent to one another. In both matrices, repetitions and intrusions were placed adjacent to one another (generally low correlation with all other scores), most switch/switch-speed scores were grouped together, and cluster/chain/edge-speed scores were grouped together.

### Regression models

#### Full complement of participants

In the all-cases analysis, WAIC for the animal base model (i.e., with raw scores, repetitions, and intrusions) was 1739.08 (see top row of Table 3). The base model showed the best performance with regard to sensitivity, negative predictive value, and F1 score. Models including cluster and switch scores calculated with the network and slope difference methods improved on the base model only marginally, with NRI 0.6%. When scores based on timings were included, those derived by partitioning with the slope difference method led to the best WAIC (1733.40), weight (0.639), and AUC (0.638), suggesting that this model might generalize best to new data points; however, the negative NRI indicates no improvement over the base model with the current data set. Other performance measures (e.g., sensitivity, specificity) were weak, hovering around 0.6.

**Table 3:** Performance of models fit with animal fluency data.

	WAIC	Weight	AUC	Sens	Spec	NPV	PPV	F1	NRI
**All participants (640 cases, 640 controls)**									
Base	1739.08	0.037	0.630	**0.630**	0.581	**0.611**	0.601	**0.615**	–
Network	1739.92	0.025	0.632	0.559	0.655	0.598	0.618	0.587	**0.006**
Troyer	1741.96	0.013	0.630	0.561	0.641	0.593	0.610	0.584	−0.011
Slope difference	1739.83	0.026	0.632	0.559	**0.661**	0.600	**0.623**	0.589	**0.006**
Network – timings	1736.81	0.116	0.635	0.559	0.650	0.596	0.615	0.586	−0.006
Troyer – timings	1736.93	0.009	0.634	0.569	0.639	0.597	0.612	0.589	−0.005
Slope dif – timings	**1733.40**	**0.639**	**0.638**	0.567	0.633	0.594	0.607	0.586	−0.008
Mayr – timings	1738.96	0.040	0.633	0.617	0.592	0.607	0.602	0.610	−0.005
**Acute cases (536 cases, 640 controls)**									
Base	1595.82	0.129	0.625	0.634	0.586	0.657	0.562	0.596	–
Network	1597.27	0.062	0.626	0.636	0.578	0.655	0.558	0.595	−0.006
Troyer	1598.32	0.037	0.625	0.647	0.569	0.658	0.557	0.599	−0.010
Slope difference	1596.18	0.108	0.626	0.655	0.569	0.663	0.560	0.604	0.001
Network – timings	1596.09	0.113	**0.630**	**0.687**	0.531	**0.669**	0.551	**0.611**	−0.003
Troyer – timings	1596.02	0.117	0.627	0.638	0.563	0.650	0.550	0.591	−0.018
Slope dif – timings	**1594.20**	**0.290**	0.628	0.627	**0.602**	0.658	**0.569**	0.596	**0.006**
Mayr – timings	1595.58	0.145	0.627	0.632	0.577	0.652	0.556	0.592	−0.009
**Progressive cases (104 cases, 640 controls)**									
Base	571.34	0.015	0.725	0.712	0.627	0.930	0.236	0.355	–
Network	573.62	0.005	0.727	0.683	0.659	0.927	0.246	0.361	0.009
Troyer	574.09	0.004	0.728	0.635	0.702	0.922	0.257	0.366	0.020
Slope difference	575.29	0.002	0.727	0.731	0.628	0.935	0.242	0.364	0.006
Network – timings	567.40	0.106	0.739	0.692	**0.703**	0.934	**0.275**	**0.393**	0.072
Troyer – timings	567.81	0.087	0.738	0.683	0.697	0.931	0.268	0.385	0.050
Slope dif – timings	**563.41**	**0.779**	**0.749**	0.788	0.638	0.949	0.261	0.392	**0.082**
Mayr – timings	574.69	0.003	0.747	**0.798**	0.625	**0.950**	0.257	0.389	0.016

Bold values represent the best performance for each metric within each analysis.

Watanabe–Akaike Information Criterion for the letter F base model was 1771.63 (see Table 4). Addition of Mayr scores improved the fit, with WAIC 1766.85, claiming 79.5% of the weight. A marginally positive NRI was observed for timing scores derived using the slope difference method (1.3%). Other performance measures were weaker than for animal fluency models, generally below 0.6.

**Table 4:** Performance of models fit with letter F fluency data.

	WAIC	Weight	AUC	Sens	Spec	NPV	PPV	F1	NRI
**All participants (640 cases, 640 controls)**									
Base	1771.63	0.073	0.590	0.613	0.552	**0.587**	0.577	0.594	–
Network	1774.57	0.017	0.590	0.589	0.561	0.577	0.573	0.581	−0.009
Troyer	1774.78	0.015	0.591	0.608	0.547	0.582	0.573	0.590	−0.009
Slope difference	1774.52	0.017	0.588	0.545	**0.623**	0.578	**0.592**	0.567	**0.013**
Network – timings	1773.41	0.030	0.594	**0.619**	0.538	0.585	0.572	**0.595**	−0.008
Troyer – timings	1774.25	0.020	**0.595**	0.567	0.586	0.575	0.578	0.573	−0.011
Slope dif – timings	1773.14	0.034	0.594	0.583	0.564	0.575	0.572	0.577	−0.020
Mayr – timings	**1766.85**	**0.795**	**0.595**	0.500	0.652	0.566	0.589	0.541	−0.009
**Acute cases (536 cases, 640 controls)**									
Base	1620.46	0.011	0.390	0.573	0.573	0.616	0.529	0.550	–
Network	1623.14	0.003	0.590	0.582	0.569	0.619	0.531	0.555	0.005
Troyer	1623.35	0.003	0.591	0.576	0.561	0.613	0.524	0.549	−0.019
Slope difference	1623.24	0.003	0.588	0.534	**0.616**	0.612	0.538	0.536	0.002
Network – timings	1622.13	0.005	0.595	0.569	0.609	**0.628**	**0.550**	0.559	**0.034**
Troyer – timings	1622.72	0.004	0.596	0.562	**0.616**	0.626	**0.550**	0.556	0.014
Slope dif – timings	1621.57	0.006	0.596	**0.597**	0.569	**0.628**	0.537	**0.565**	0.009
Mayr – timings	**1611.48**	**0.967**	**0.601**	0.562	0.577	0.611	0.526	0.543	−0.002
**Progressive cases (104 cases, 640 controls)**									
Base	591.87	0.115	0.670	**0.731**	0.542	**0.925**	0.206	0.321	–
Network	**588.04**	**0.783**	**0.685**	0.683	0.613	0.922	0.223	**0.336**	**0.051**
Troyer	596.07	0.014	0.671	0.615	0.634	0.910	0.215	0.318	−0.004
Slope difference	594.42	0.032	0.676	0.663	0.598	0.916	0.212	0.321	−0.004
Network – timings	595.60	0.018	0.671	0.615	**0.653**	0.913	**0.224**	0.328	0.005
Troyer – timings	596.25	0.013	0.671	0.625	0.627	0.911	0.214	0.319	−0.018
Slope dif – timings	596.52	0.011	0.669	0.635	0.614	0.912	0.211	0.317	−0.023
Mayr – timings	596.25	0.013	0.672	0.721	0.552	0.924	0.207	0.322	0.018

Bold values represent the best performance for each metric within each analysis.

#### Acute decliners

For acute decliners, the WAIC for the base model trained with animal fluency data was 1595.82. As with the models trained on the full complement, the model trained with speed scores derived using the slope difference algorithm led to marginal improvement in WAIC (1594.20) and received 29.0% of the weight among the compared models. This model also had a marginally positive NRI (0.6%).

Letter F WAIC for the base model comparing acute decliners to controls was 1620.46. Mayr scores again improved the fit of the model, which claimed 96.7% of the weight (WAIC 1611.48). The best NRI resulted from addition of network-derived speed scores (3.4%).

#### Progressive decliners

For progressive decliners, the WAIC for the base model trained with animal fluency data was 571.34. Incorporation of timing-based scores derived from slope difference led to a substantial improvement in WAIC (563.41) and this model received 77.9% of the weight among the compared models. This model had the best AUC (0.749) and NRI (8.2%).

Letter F WAIC for the base model comparing progressive decliners to controls was 591.87. The model incorporating network-derived clustering and switching scores led to slight improvement in the WAIC (588.04), such that this model was allocated 78.3% of the weight and the base model received 11.5%. The best NRI score was observed with the network model (5.1%).

#### Combined models

Posterior distributions for logistic regression models combining switch and edge speed scores with the letter fluency base model were calculated using Markov chain Monte Carlo analysis. Table 5 shows means and 97% HDI for each variable in the model fit to all participants, comparing acute decliners to controls, and comparing progressive decliners to controls. Posterior histograms for all-cases, acute-cases, and progressive-cases analyses are shown in Supplementary Figures S1–S3, respectively. Coefficients for the animal raw, switch speed, and edge speed scores are larger in magnitude (more negative) in the progressive-cases model than for the acute-cases or all-cases models. The opposite pattern holds for letter F raw scores, where the coefficients have a larger magnitude among acute-cases and all-cases models. Coefficients for the demographic variables were generally different from the effects observed in the univariate analysis. See the Discussion for more details about these findings.

**Table 5:** Mean and 97% highest density interval (HDI) of combined model posterior distributions.

Variable	All participants		Acute		Progressive	
	Mean	HDI	Mean	HDI	Mean	HDI
Intercept	2.707	(1.218, 4.105)	2.954	(1.442, 4.465)	−0.817	(−3.735, 2.064)
Animal SD Switch speed	−0.315	(−0.55, −0.08)	−0.248	(−0.49, −0.007)	−0.793	(−1.312, −0.292)
Animal SD Edge speed	−0.221	(−0.418, −0.019)	−0.176	(−0.382, 0.03)	−0.531	(−0.958, −0.12)
Animal raw	−0.079	(−0.11, −0.049)	−0.07	(−0.102, −0.039)	−0.165	(−0.234, −0.096)
Animal repeats	0.073	(−0.019, 0.166)	0.072	(−0.023, 0.168)	0.078	(−0.096, 0.252)
Animal intrusions	0.257	(−0.195, 0.727)	0.303	(−0.151, 0.779)	−0.107	(−1.157, 0.882)
Letter F switch	–	–	–	–	−0.167	(−0.555, 0.235)
Letter F cluster	–	–	–	–	−1.195	(−3.375, 0.874)
Letter F Mayr C	0.036	(−0.013, 0.085)	0.046	(−0.006, 0.099)	–	–
Letter F Mayr S	0.015	(−0.067, 0.096)	0.011	(−0.084, 0.104)	–	–
Letter F – raw	−0.041	(−0.075, −0.006)	−0.045	(−0.081, −0.008)	−0.015	(−0.082, 0.053)
Letter F – repeats	0.079	(−0.018, 0.179)	0.053	(−0.053, 0.158)	0.192	(0.024, 0.369)
Letter F – intrusions	0.091	(−0.083, 0.263)	0.086	(−0.09, 0.266)	0.101	(−0.268, 0.455)
Age	−0.022	(−0.039, −0.006)	−0.03	(−0.047, −0.012)	0.008	(−0.024, 0.042)
Male	0.03	(−0.235, 0.289)	0.117	(−0.151, 0.393)	−0.471	(−1.012, 0.058)
HS	0.084	(−0.36, 0.528)	0.188	(−0.297, 0.649)	−0.473	(−1.324, 0.39)
Some college	0.229	(−0.225, 0.697)	0.25	(−0.238, 0.744)	0.049	(−0.789, 0.884)
College grad	0.365	(−0.102, 0.823)	0.417	(−0.081, 0.913)	0.167	(−0.699, 1.007)
White	0.35	(0.067, 0.648)	0.287	(−0.013, 0.59)	0.858	(0.263, 1.486)
Belt	−0.131	(−0.426, 0.159)	−0.164	(−0.466, 0.142)	0.128	(−0.442, 0.702)
Buckle	−0.12	(−0.461, 0.222)	−0.151	(−0.509, 0.21)	−0.084	(−0.77, 0.58)

HDI, 97% highest density interval; HS, high school education; Mayr C, intercept term from regression model; Mayr S, slope term from regression model; SD, slope difference.

Union of the variables in the best animal and letter F models led to an improvement in WAIC in the all-cases and acute-cases analyses (see Table 6). For the all-cases analysis, the combined model claimed 94% of the weight and exhibited NRI of 0.9%. For the acute-cases analysis, the combined model claimed 98.5% of the weight and exhibited NRI of 1.3%. For the progressive decliners analysis, the model with animal fluency scores (slope difference speed scores) remained superior to the combined model (77.7% *vs*. 20.9% of the weight, and 8.2% *vs*. 7.2% NRI).

**Table 6:** Comparisons of combined models.

	WAIC	Weight	AUC	Sens	Spec	NPV	PPV	F1	NRI
**All cases**									
Base	1739.08	0.004	0.630	**0.630**	0.581	**0.611**	0.601	**0.615**	–
Animal slope dif – timings	1733.40	0.057	0.638	0.567	0.633	0.594	0.607	0.586	−0.008
+ Letter F Mayr	**1728.16**	**0.940**	**0.650**	0.584	**0.641**	0.607	**0.619**	0.601	**0.009**
**Acute cases**									
Base	1595.99	0.005	0.625	**0.634**	0.586	0.657	0.562	0.596	–
Animal slope dif – timings	1594.33	0.011	0.628	0.627	0.602	**0.658**	0.569	0.596	0.006
+ Letter F Mayr	**1585.29**	**0.985**	**0.646**	0.614	**0.622**	**0.658**	**0.576**	**0.594**	**0.013**
**Progressive cases**									
Base	571.69	0.014	0.725	0.712	0.627	0.930	0.236	0.355	–
Animal slope dif – timings	**563.68**	**0.777**	0.749	**0.788**	0.638	**0.949**	0.261	0.392	**0.082**
+ Letter F Network	566.30	0.209	**0.756**	0.702	**0.698**	0.935	**0.274**	**0.395**	0.072

Bold values represent the best performance for a specific metric (column) within a given analysis. WAIC, Watanabe–Akaike Information Criterion; AUC, area under the ROC curve; Sens, sensitivity; Spec, specificity; NPV, negative predictive value; PPV, positive predictive value; F1, harmonic mean of specificity and PPV; NRI, net reclassification improvement.

## Discussion

We compared three methods for identifying related items in animal and letter fluency word lists. The correspondence between pairs of techniques (Cohen’s κ) was relatively higher for animal fluency than for letter fluency. For both tasks, the correspondence was highest between the traditional Troyer method and the network-based method. Importantly, the lack of correspondence between slope difference and the other methods does not necessarily imply a weakness of any technique. It does appear that the information imparted by the slope difference technique is different from the information imparted by the other two. Correlations were generally high between clustering scores derived by different methods and between switching scores derived by different methods. The largest negative correlations tended to be between clustering and switching scores, consistent with their known complementary nature. The Mayr constant and slope scores showed the weakest correlations with the other scores. This finding is not terribly surprising, as they rest on a theoretical foundation that is distinctly different from that underlying clustering and switching scores.

Participants generated twice as many F-word types as animal types (867 *vs*. 433). Comparison of the two networks suggests that the letter F network was not only larger (more vertices and edges), but also more sparse. For letter F, 6% of possible edges were present, while for animals, 11% of possible edges were present. The letter F network clustering coefficient was also much lower. We would therefore expect specific pairs of consecutive words (bigram tokens) to occur more frequently with random walks on the animal graph than on the letter F graph. To the extent that these network models reflect true qualities of the mental lexicon in English-speaking Americans, their differing topographies may be considered an explanation for the apparent insufficiency of the Goñi method for building the letter F network. We conjecture that despite our generous sample size, the sparse nature of the letter F network entails the need for a much larger corpus of verbal fluency lists to identify edges in a statistically reliable way.

We further compared models trained with different verbal fluency scoring techniques according to their capability to identify subjects who would experience future cognitive decline. For each fluency task, we began with a base model that included only demographic variables, traditional raw scores, repetitions, and intrusions. We then assessed the value of different techniques for calculating clustering, switching, and speed scores by entering the scores into logistic regression models with the outcome variable of ICI.

Speed scores measured during the animal task between related words (edge transitions) and unrelated words (switch transitions) may have value for identifying individuals at high risk for imminent, progressive decline in cognition (see lower third of Table 3). We speculate that, at least in the case of animal fluency, this finding could reflect degeneration of neurons in the lexical-semantic network, resulting in reduced rate of spreading activation. It is less clear whether declines in switch speed are better conceptualized as reduced activation of animal subcategories or as dysfunction of executive processes necessary for word retrieval. We note that words starting with F cannot be easily placed into a few distinct subcategories, while participants and researchers have little difficulty placing animal words into such subcategories. This simple observation about the two tasks meshes well with the differences in network topography described previously. Perhaps, future work will establish the relative importance of semantic and executive skills for efficiently searching such networks. We emphasize that Mayr (2002) suggests that the cluster/switch dichotomy is potentially erroneous because it fails to differentiate between a general reduction in processing speed and a specific deficit of switching. While we find that scores derived by applying Mayr’s technique to letter fluency timings seem to aid in the empirical detection of individuals at increased risk for acute cognitive decline, our current findings do not exclude the possibility that classic clustering and switching scores could be valuable for other kinds of clinical distinctions (e.g., distinguishing between specific forms of neuropathology) or could make valuable contributions to more comprehensive machine learning models, which may incorporate numerous other predictive features as well as the myriad interactions among them. Our findings offer a few recommendations for choosing scores for such models, however. First, the slope difference method shows little overlap with the network and Troyer methods, suggesting that scores derived using slope difference may complement these other methods. Second, speed scores may be superior to classic clustering and switching scores (i.e., more sensitive indicators of the earliest cognitive changes). Third, animal fluency scores appear to be superior to letter fluency scores for identifying individuals at high risk for cognitive impairment in general. This observation holds true, especially for our “progressive” group. We have argued elsewhere that this group is likely to consist mainly of individuals with AD, while the acute group is likely to contain individuals with other pathologies (e.g., vascular disease or Lewy body diseases) as well as individuals with AD, albeit at an earlier stage of disease (Ayers et al., 2022).

When studying patients with degenerative brain diseases, such as AD, one might expect variation in cognitive measures to reflect differences in the topography of cerebral pathology. For example, it is known that in AD, tau pathology spreads from medial temporal regions to neocortical regions, including the lateral temporal lobe (Braak and Braak, 1991). Involvement of this region by tau pathology could affect clustering scores in a manner like what has been described with focal lesions. This prediction finds some support in the literature, as patients with AD exhibit reductions in clustering, especially with semantic fluency tasks (Tröster et al., 1998; Gomez and White, 2006; Weakley et al., 2013). Similarly, switching scores may be impacted by neuropathologic involvement of frontal-subcortical circuits. Reductions in switching scores have been reported in dementia due to Parkinson’s disease (PD; Tröster et al., 1998; Troyer et al., 1998b), Huntington’s disease (Ho et al., 2002), vascular dementia (Zhao et al., 2013), and in patients with PD who have undergone pallidotomy (York et al., 2003).

These cognitive, anatomic, and pathological distinctions raise the question of whether patterns of qualitative performance on verbal fluency might contribute meaningfully to prediction or early detection of cognitive impairment. For example, a decline in clustering scores could herald the earliest spread of tau into temporal neocortical regions supporting word knowledge (Dronkers et al., 2004). A few lines of evidence support this conjecture. Patients with AD produce smaller clusters than controls on animal fluency, with amnestic MCI patients falling between the two groups, suggesting that the transition to dementia is accompanied by declining clustering score (Murphy et al., 2006). Apolipoprotein E ε4 carriers, who are known to be at increased risk for AD, have lower mean clustering scores than non-carriers and take longer to access those clusters (Rosen et al., 2005). Memory-impaired individuals with lower clustering scores appear to be more likely to develop AD (Fagundo et al., 2008). Similarly, higher average cluster size is associated with reduced risk of dementia over the ensuing years among volunteers in the Nun’s Study (Pakhomov et al., 2012). Apart from clustering scores, there are other scores based on semantic relatedness in fluency word lists that have putative prognostic value (Pakhomov et al., 2016).

Alzheimer’s disease is not classically associated with pathology in frontal-subcortical circuits, but patients with AD frequently exhibit executive dysfunction (Swanberg et al., 2004). Thus, one may hypothesize that switching scores may decline during the transition to AD dementia. This hypothesis finds support in a few studies in which switching scores appear to be more valuable than mean cluster size for prediction of MCI conversion to dementia or other measures of functional decline (Raoux et al., 2008; Clark et al., 2014, 2016). These findings replicate other work underscoring the important relationship between decline in executive skills and loss of independent daily function in AD (Albert, 1996).

With one exception, models incorporating mean cluster size and switch counts did not lead to substantial improvement over base models. The exception was the model using the network method for letter fluency, but the improvement was seen only in the progressive cases analysis (NRI 5.1% – see lower third of Table 4). The WAIC for this model was better than that of the base model (588.04 vs. 591.87, with weights of 78.3% and 11.5%, respectively). Other investigators have reported statistically significant contributions of clustering (Pakhomov and Hemmy, 2014) or switching (Raoux et al., 2008) scores calculated from animal fluency for the prediction of dementia. Importantly, Pakhomov and Hemmy (2014) included an alternative outcome measure of incident *memory* impairment (a more subtle distinction than dementia), but did not identify a significant contribution of clustering for this outcome. The SIS score, which consists only of three delayed recall items and three temporal orientation items, is arguably more similar to the memory outcome measure of Pakhomov and Hemmy (2014) than to the dementia outcome. Thus, our finding that animal fluency clustering scores do not improve on a base model for predicting ICI replicates this negative finding.

The slope difference approach to identifying links stands out for a few reasons. Among the data-driven techniques, it is the only one that relies entirely on data intrinsic to individual word lists, rather than an external corpus. For both fluency tasks, the agreement between slope difference and the other two methods was relatively low, indicating that the slope difference algorithm is not merely replicating the other methods. However, for all three animal fluency analyses, the model incorporating slope difference speed scores achieved the best WAIC.

Comparison of the posterior distributions for the combined models of the three analyses yields a few interesting insights. First, the 97% HDI excludes 0 for all animal fluency raw and speed scores in the three combined models, with one exception: animal fluency edge transition speed appears more weakly associated with acute decline than with progressive decline. This raises the question of whether individuals in the acute decliner group maintain some capacity for rapidly following strong associative links, either because they differ pathologically from the progressive group or because they were observed earlier during the trajectory toward progressive dementia. Second, the 97% HDI for letter F raw score is reliably negative and excludes 0 in the acute decliner analysis, but is very weak (*β* = −0.015) in the progressive decliner analysis. Given our two hypotheses about the distinctions between these two groups, this finding seems to give greater support to the idea that at least some of the acute decliners are suffering from a different pathological process than the progressive decliners, as it seems unlikely (though not impossible) that the progressive decliners have passed through a temporary phase of increased difficulty with letter fluency and then emerged from it. Third, the 97% HDI for letter fluency repetitions excludes 0 only for the progressive decliners. This finding suggests either greater memory impairment or greater difficulty inhibiting unwanted responses among the progressive group. Either phenomenon would fit with our hypotheses about the progressive decliners, but the former interpretation fits better with the observation that the HDIs for both animal intrusions and letter F intrusions are quite broad in this group. In contrast, nearly all of the probability density of the HDI for letter F intrusions is positive in the acute-cases analyses, raising the question of relatively greater difficulty with inhibition of unwanted responses in this group. Thus, we tentatively conclude that the progressive group has greater memory impairment while the acute group has greater difficulty inhibiting unwanted responses. Fourth, while addition of the Mayr scores improves the acute cases model, we note that the Mayr C shows a stronger relationship with ICI than the S term (*β* = 0.046 vs. 0.011). We propose that the greater difficulty of the letter fluency task makes it more sensitive than animal fluency to these non-semantic factors and that the C term provides a measure of subtle executive dysfunction that relates to increased risk for acute decline in cognition. Fifth, the findings for some demographic variables (age, race, and educational level) are not intuitive and differ somewhat from the univariate analyses presented in Table 1. In the univariate comparisons, it appears that age differs between cases and controls only in the progressive decliners comparison (log BF 0.85, but < −1.0 for acute and all-cases groups). However, the posteriors for the combined models suggest a protective effect of increasing age, except in the progressive cases model. Our interpretation for this finding is that these demographic variables are weaker predictors of ICI than the verbal fluency scores included in each model. Taking age as an example, imagine two hypothetical individuals of different ages, but with identical verbal fluency performance. We would consider the verbal fluency scores of the older individual to be relatively better than those of the younger individual, and this difference could translate into lower risk for ICI. In the progressive decliners model, the relationship between age and ICI seems to evaporate with inclusion of the verbal fluency scores (*β* = 0.008), suggesting that the relationship between age and ICI could be mediated by verbal fluency performance. Perhaps such findings should be expected because the cases and controls were matched on these demographic variables. We would invoke similar reasoning to explain the counterintuitive relationships between ICI and other demographic variables. However, we do not see such an inversion in the observed effect of sex between the univariate comparisons and the regression models. In both the univariate and regression analyses, male sex appears to be protective against progressive cognitive decline. This finding is in keeping with other results suggesting that women are at increased risk of AD (Payami et al., 1994; Clark et al., 2018). Finally, we note that the posterior probability distributions for these models could serve as priors for future Bayesian analyses of the questions we attempt to address here.

The main limitation of this study is the heterogeneity of the ICI cohort. We do not know the actual diagnoses of subjects who developed cognitive impairment, only that the absence of clinical stroke in a population exhibiting cognitive decline makes AD seem to be a likely explanation for a majority of cases. More optimistically, the long duration of follow-up (nearly 9 years) gives strong support to the view that our controls were cognitively normal at the time of the verbal fluency recordings. In future work, imaging data and biomarkers would mitigate the concern of heterogeneity, and perhaps could yield a more refined evaluation of clustering and switching techniques. Although the Bayesian logistic regression we employed imparts certain advantages (enumerated in the Methods), it does not examine potential nonlinear relationships between the outcome and predictors, nor can it account for the myriad potential interactions among the predictors. We have strived to explore a broad range of techniques designed for measuring the cognitive processes underlying verbal fluency, but we acknowledge that this work is not comprehensive and other approaches (e.g., optimal foraging, diffusion drift) may yield new insights in future work (Hills et al., 2012; Kakkar, 2020; Wadhera and Kakkar, 2020).

## Conclusion

For animal fluency, we find that models including scores based on participant speed outperform the base model. We observe the most unambiguous improvement in a model discerning between controls and individuals with progressive decline, and including speed scores derived using the slope difference method. For letter fluency, models outperforming the base model differ between the acute and progressive decliner groups. A score indexing executive contributions to letter fluency improves the fit for the acute decliners, while a clustering score derived from a network-based determination of word linkages improves the fit and NRI of the model for progressive decliners. Combining the best animal and letter fluency models leads to further improvement in model fit for the all-cases and acute-cases models, but not to substantial increases in NRI. Automatic transcription and timing of fluency word lists may prove valuable as a widely accessible and potentially remotely administered approach to stratifying individuals in terms of risk for incident cognitive impairment.

## Data availability statement

The data analyzed in this study is subject to the following licenses/restrictions: Analysis of data from the Reasons for Geographic and Racial Differences in Stroke (REGARDS) study requires review by the REGARDS Executive Committee. Publication of results requires approval by the REGARDS Manuscript Committee. Requests to access these datasets should be directed to DC, clarkdg@iu.edu.

## Ethics statement

The studies involving human participants were reviewed and approved by the Institutional Review Boards of the University of Alabama at Birmingham and Indiana University. The patients/participants provided their written informed consent to participate in this study.

## Author contributions

JB penned original manuscript and critical contribution to analyses and presentation. DS contributed to network analysis. MA contributed to conceptualization and manuscript review. SG advised on statistical analysis. FU contributed to fundamental study design and manuscript review. JD developed custom software for verbal fluency transcription. JG developed software for network analysis, and advised on network approach. VW contributed to fundamental study design and manuscript review. RK contributed to fundamental study design and manuscript review. DC conceptualized original approach, aided in manuscript development, and performed statistical analysis. All authors contributed to the article and approved the submitted version.

## Funding

This work was supported by National Institutes of Health grants to the REGARDS Study (NIH U01 NS041588) and to the Indiana University Alzheimer Disease Research Center (NIH P30 AG010133 and P30 AG072976).

## Conflict of interest

The authors declare that the research was conducted in the absence of any commercial or financial relationships that could be construed as a potential conflict of interest.

## Publisher’s note

All claims expressed in this article are solely those of the authors and do not necessarily represent those of their affiliated organizations, or those of the publisher, the editors and the reviewers. Any product that may be evaluated in this article, or claim that may be made by its manufacturer, is not guaranteed or endorsed by the publisher.

## Footnotes

^1^The set of associations described for the Troyer technique, consisting of a list of subcategories and specific exemplars within each subcategory, cannot be represented as a simple network. When following Troyer’s technique, a cluster is defined as a sequence of words *all belonging to the same subcategory*. Thus, to represent the Troyer data as a network would require a generalization of graphs known as a *multigraph*, in which a pair of vertices may be linked by multiple edges. Such edges may be separated into types and labeled, for example, with different colors. Words constituting a cluster must map to a set of vertices that form a complete subgraph with edges of uniform color.
