# Supplementary material for: A comparison of techniques for deriving clustering and switching scores from verbal fluency word lists
Source: Front Psychol. 2022 Sep 14;13:743557. doi: 10.3389/fpsyg.2022.743557 (PMC9518694; doi:10.3389/fpsyg.2022.743557)
Supplement: Supplementary file 1 [file Table_1.pdf]

Supplementary Table e-1. Animal subcategories and specific exemplars

rabbit	bunnies, rabbits, pika, coneys, hare, bunny_rabbit, coney, rabbit, jackrabbit, mammal, bunny
micro-organism	protozoa, ameba, virus, bacteria, amoeba, phytoplankton, paramecium, zooplankton, euglena, nematode
insects	firefly, flies, ticks, earthworm, beetles, insects, wasp, wood_louse, grasshopper, spider, ants, housefly, ant, wasps, beetle, fireflies, katydid, gnats, earthworms, gnat, tick, preying_mantis, cockroaches, bed_bugs, scorpion, flower_beetle, butterflies, lady_bug, moth, bed_bug, bees, caterpillars, bug, crickets, mosquitoes, leech, butterfly, ladybug, cricket, palmetto_bug, snail, chiggers, locusts, termite, flew, worms, caterpillar, palmetto_bugs, cockroach, chigger, flea, dung_beetle, roach, mite, roaches, slug, fly, fleas, louse, insect, wood_lice, lice, pillbug, locust, worm, bee, roly_poly, centipede, flown, bugs, mosquito, sow_bug, stag_beetle, snails, grasshoppers, tarantula, crane_fly, hornet, honey_bee, bumblebee, yellow_jacket, earwig, horsefly
fish	perch, tilapia, mackerel, placoderm, guppy, sunfish, mahi-mahi, anchovies, herring, piranha, flounder, sawfish, manta_ray, bluegill, trout, anchovy, bass, salmon, carp, swordfish, bream, goldfish, tuna, barracuda, tarpon, seahorse, catfish, eel, placoderms, halibut, red_snapper, walleye, stingray, grouper, minnow, cod, panfish, suckerfish, zebrafish, pike, brown_trout, jellyfish, tench, sand_dab, shellfish.sailfish, marlin
deer	fawn, roebuck, antelope, elands, elks, elk, eland, gazelle, wildebeest, oryx, reindeer, mule_deer, moose, fallow_deer, kudus, kudu, gnus, buck, antelopes, gnu, caribou, doe, mammal, gazelles, impala, stag
canine	schnauzers, yorkie_terrier, cockers, greyhound, foxes, yorkie, cocker, mutt, collies, hound, pointer, panda, boston_bull, chihuahua, pomeranian, coyotes, pups, fox, poodle, dachshund, beagle, poodles, jackals, puppy, pandas, jackal, collie_dogs, great_dane, bulldog, eskimo_dog, wild_dogs, spaniel, mini_bulldog, canine, mammal, coyote, wild_dog, pup, shih-tzu, collie_dog, schnauzer, hound_dog, dingo, canines, dog, hyena, wolf, pitbull, cocker_spaniel, puppies, wolves, german_shepherd, hound_dogs, collie, chow_chow, bloodhound, basset_hound, mastiff, foxhound, vixen
bovine	oxen, plain_buffalo, cattle, cows, guernsey_cow, cape_buffalo, milk_cow, yak, holstein_cow, musk_ox, calf, buffalos, jersey_cow, bulls, water_buffalo, bull, milk_cows, beef_cow, calves, cow, heifer, bison, mammal, buffalo, steer, angus, perissodactyla, artiodactyla, muskox
rodent	rats, chipmunk, marmot, gopher, rodent, rat, gophers, beaver, mouse, mole, muskrat, gerbil, guinea_pig, white_rat, hyrax, hamsters, tree_shrew, woodchuck, capybara, ground_squirrel, cavy, guineas, guinea_pigs, bunny, chinchilla, porcupine, squirrel, chipmunks, coendu, flying_squirrel, white_rats, tree_squirrel, mice, squirrels, groundhogs, beavers, prairie_dog, hamster, guinea, mammal, groundhog, moles, rock_hyrax, hedgehog, red_squirrel, nutria, lemming, kangaroo_rat, pack_rat
weasel	minks, weasel, ferrets, wolverine, ferret, marten, polecat, mongoose, badger, mammal, skunk, mink, otters, meerkat, otter, ermine, honey_badger, civet, fisher
porcine	hog, wild_boar, piglet, sows, pig, hogs, pigs, sow, piglets, swine, perissodactyla, artiodactyla

extinct	brachiosaurus, dodo_bird, placoderm, dinosaur, placoderms, mammoth, woolly_mammoth, saber_toothed_cat, tyrannosaurus, pterodactyl
nocturnal	owl, bat, screech_owl, bats, owls, kangaroo_rat, civet
unusual-mammal	ant_bear, sloth, bat, tapir, giant_ground_sloth, koala_bear, three-toed_sloth, coendu, killer_whale, great_white_whale, armadillos, pilot_whale, ringtail_lemur, wallaby, bats, wallabies, california_whale, armadillo, flying_lemur, two-toed_sloth, tamandua, orca, sealion, coati, pinniped
insectivore	shrew, ant_bear, aardvark, mole, anteater, tamandua, moles, hedgehog
pet	cockatoo, schnauzers, yorkie, pony, yorkie_terrier, cockers, golden_retriever, canary, hamsters, cocker, mutt, irish_setter, collies, rabbits, hound, guinea_pig, dachshund, pointer, boston_bull, chihuahua, gerbil, parrots, kitty_cats, pups, arabian_horse, poodle, beagle, bulldog, guinea, cats, collie_dogs, cavy, guineas, guinea_pigs, puppy, canaries, bunny, cockatiel, paso_fino_horse, pomeranian, kittens, budgie, greyhound, ponies, pitbull, kitty, eskimo_dog, spaniel, mini_bulldog, peruvian_horse, rabbit, kitten, goldfish, hound_dog, terrapin, pup, poodles, shih-tzu, collie_dog, schnauzer, hound_dogs, bunnies, great_dane, tomcat, parrot, kitty_cat, boa_constrictor, shetland_pony, dog, cat, cockateel, shih_tzu, hamster, lorikeet, cocker_spaniel, puppies, german_shepherd, kitties, collie, chow_chow, house_finch, garter_snake, zebrafish, siamese_cat, burmese_python, sphynx, basset_hound, calico_cat, mastiff, budgerigar, foxhound, gila_monster
feline	bengal_tiger, snow_leopard, cougars, lion, ocelot, bobcat, mountain_lion, puma, panther, kitty_cats, bengal, cougar, cats, lions, liger, cheetah, cheetahs, pumas, ocelots, kittens, kitty, leopard, tiger, civet_cat, jaguars, kitten, wildcat, jaguar, tomcat, siberian_tiger, cat, wildcats, lynx, kitty_cat, kitties, siamese_cat, sphynx, calico_cat, saber_toothed_cat, catamount, jaguarundi, margay
small-wild-mammal	racoon, wolverine, squirrel, raccoons, opossums, marmot, kudamonday, armadillos, marmots, possum, opossum, armadillo, raccoon, bandicoot, squirrels, red_squirrel, civet
australian	kiwi, wombat, koala, koala_bear, dingo, tasmanian_devil, kangaroo, platypus, emu, opossum, koala_bears, wallabies, wallaby, kiwis, kangaroos, komodo_dragon, kookaburra, kangaroo_rat, echidna
reptile-amphibian	turtle, brachiosaurus, cobra, serpent, bullfrog, reptile, terrapin, crocodile, anaconda, gecko, croc, iguana, tadpoles, alligator, coral_snake, copperhead, rattlesnake, crocodiles, tadpole, reptiles, serpents, salamanders, water_moccasin, chameleon, toad, salamander, amphibians, alligators, newt, lizards, tortoise, turtles, boa_constrictor, snake, dragon, dinosaur, lizard, amphibian, python, adder, garter_snake, diamondback_rattlesnake, cottonmouth, burmese_python, african_rock_python, timber_rattlesnake, viper, blacksnake, skink, horntoad, komodo_dragon, tyrannosaurus, pterodactyl, gila_monster, caecilian
farm	ass, donkey, pony, piglet, turkeys, turkey, sows, regular_pig, ram, pig, goose, hogs, ducklings, sow, duck, cows, oxen, chicken, hens, equines, guernsey_cow, sheep, hog, horse, lambs, cattle, milk_cow, capon, goats, ferrets, asses, goat, geese, jackass, colt, calf, horses, lamb, plow_horse, ferret, ponies, mule, fowl, billy_goat, mules, milk_cows, sheeps, rooster, colts, hen,

	equine, calves, fowls, cow, heifer, shetland_pony, duckling, chickens, filly, rams, beef_cow, holstein_cow, jersey_cow, donkeys, piglets, steer, bantam, chick, mustang, stallion, guineafowl, onager, mare, swine, foal, angus, perissodactyla, artiodactyla
arctic	walrus, auk, polar_bears, polar_bear, reindeer, seal, walruses, seals, caribou, musk_ox, penguin, lemming, muskox, pinniped
bear	black_bears, kodiak_bear, pandas, grizzly, panda_bear, grizzly_bear, brown_bear, black_bear, cub, mammal, golden_bear, panda
water	mahi_mahi, turtle, shark, clams, tilapia, animals_from_sea, perch, frogs, fish, dolphin, great_white_whale, seals, crocodile, otters, red_snapper, lobster, seal, california_whale, beaver, herring, whale, muskrat, mahi-mahi, crocodiles, whales, crawfish, auk, killer_whale, gull, crustaceans, frog, newt, flounder, salamanders, catfish, crab, manta_ray, bluegill, penguin, sea_lion, tuna, squid, crustacean, starfish, toad, mussels, carp, swordfish, salamander, hermit_crab, clam, manatee, bream, sardines, fishes, alligators, pilot_whale, seagulls, krill, oyster, limpet, crabs, dolphins, otter, sardine, porpoises, turtles, beavers, oysters, jelly_fish, platypus, shrimp, alligator, leech, eel, mussel, seagull, crayfish, octopus, porpoise, walleye, stingray, grouper, minnow, jellyfish, loon, tench, mallard, sand_dab, shellfish, sailfish, barnacle, abalone, orca, marlin, sealion, sea_urchin, pinniped
ovine	sheep, lamb, billy_goats, musk_goat, nanny_goat, ram, ewe, rams, millie_goat, musk_goats, billy_goat, dall_sheep, mountain_goat, goat, sheeps, goats, lambs, mountain_sheep, perissodactyla, artiodactyla, ibex
burden	horses, donkey, oxen, camel, colt, plow_horse, pony, race_horse, equines, horse, alpaca, asses, paso_fino_horse, jackass, ass, vicuna, ponies, llamas, mule, burro, peruvian_horse, llama, mules, colts, dromedary, arabian_horse, equine, shetland_pony, filly, camels, ox, donkeys, guanaco, steer, mustang, stallion, onager, mare
hunted	rabbits, grouse, fowl, ram, deer, foxes, plain_buffalo, goose, elks, duck, boar, quail, doe, tilapia, quails, wild_boar, mahi-mahi, fox, squab, bison, geese, elk, boars, fowls, rabbit, partridge, tuna, dove, buck, grizzly_bear, rams, mammoth, doves, caribou, pheasant, stag, guineafowl, vixen
america	sloth, rabbits, chipmunk, deer, foxes, plain_buffalo, elks, pumas, wolf, beaver, bobcat, two-toed_sloth, tamandua, elk, puma, coyotes, fox, armadillos, opossum, capybara, chipmunks, badger, moose, tapir, squirrel, raccoons, coendu, manatee, possum, armadillo, bear, rabbit, three-toed_sloth, coyote, squirrels, racoon, guanaco, beavers, prairie_dog, raccoon, skunk, caribou, loon, mallard, red_squirrel, stag, vixen, ermine, fisher
raptor	screech_owl, eagle, kite, turkey_vulture, owl, falcons, hawks, vulture, buzzard, owls, birds_of_prey, bald_eagle, eagles, falcon, vultures, hawk, osprey, redtail_hawk, raptor, buzzards, red_tailed_hawk
fur	sheep, mink, alpaca, beavers, fox, foxes, minks, chinchilla, rabbit, beaver, mammal, rabbits, guanaco, sheeps, vixen, muskox
mythical	unicorns, unicorn
intrusion	force, family, facility, avocado, pic, farrel, gernic, flush, facts, ship, floury, gernik, mutton, peanut, fix, flunk, ships, lamp, fight, tail, fought, fela, flowing, furor, shrink, musk, ball, flight, corn, herd, teddy_bears, flag, fruit, picadilly, ear, fush, box, wanda,

	fustoubin, light, flow, albinos, pansy, jungle, teddy_bear, flint, albino, ping, dare, fact
primate	macaque, baboons, indri, people, chimp, jackaroo, human, ringtail_lemur, girl, children, ape, monkey, kudamonday, human_being, girls, chimps, monkeys, macaques, flying_lemur, woman, humans, men, chimpanzee, human_beings, child, chimp_monkey, gibbons, man, shrew, baboon, lemur, great_ape, marmoset, gorilla, women, native, orangutan, lemurs, mammal, gibbon, natives, bonobo, mandrill
bird	peacock, hummingbirds, redtail_hawk, sparrow, pigeons, parakeets, canary, ducklings, falcon, birds, kite, condor, swan, gull, chickadee, wren, parakeet, turkey_vulture, budgie, vulture, partridge, tern, flamingo, parrot, big_bird, red_bird, crane, sparrows, cockatoo, grouse, crow, goose, chickadees, storks, pigeon, capon, macaws, kiwis, bird, eagle, bluebirds, sardines, raptor, pelicans, bald_eagle, lorikeet, swallow, owl, roadrunner, macaw, hawks, buzzard, bluebird, stork, eagles, hen, hummingbird, cockatiel, kiwi, toucan, big_birds, osprey, penguin, bluejay, redbird, red_birds, blackbird, seagull, raven, pelican, robin, geese, buzzards, fowl, rooster, mockingbird, woodpecker, hens, egret, meadowlark, cardinal, squab, finch, canaries, seagulls, dodo_bird, fowls, blue_heron, great_auk, hawk, california_jay, screech_owl, sardine, quetzal, peacocks, duckling, cockateel, parrots, house_finch, antbird, blue_footed_booby, albatross, nightjar, bunting, nightingale, secretary_bird, frigatebird, red_tailed_hawk, grackle, loon, towhee, goldfinch, mallard, bantam, chick, starling, budgerigar, nuthatch, rhea, oriole, kookaburra, guineafowl, ibis
african	antelope, gorilla, indri, elands, chimp, cobra, lion, zebra, mongoose, warthog, jackal, hippopotamus, kudus, eland, monkey, panther, gazelle, cheetahs, camel, wildebeest, ostriches, chimps, rhino, rhinoceros, lions, elephant, cheetah, jackals, cape_buffalo, hippo, chameleon, buffalos, leopard, chimpanzee, tiger, giraffe, manatee, hippos, kudu, gnus, monkeys, ostrich, ant_bear, flamingo, antelopes, gnu, buffalo, aardvark, elephants, hyena, dromedary, oryx, camels, giraffes, lemurs, gazelles, impala, lemur, meerkat, white_rhinoceros, saber_toothed_cat, bonobo, mandrill, okapi, perissodactyla, artiodactyla, honey_badger, ibex, civet, pachyderm
asian	snow_leopard, bengal_tiger, siberian_tiger, panda_bear, tree_shrew, bengal, dragon, ringtail_lemur, flying_lemur, honey_badger
south-american	rhea, jaguarundi, vicuna, margay, llama, capybara, macaw, alpaca, tapir, peccary, chinchilla, jaguar, coatimundi, coati, flamingo, nutria, javelina
marsupial	kangaroo, echidna, opossum, wallaby, koala, sugar_glider, wombat, bandicoot

Note: the “intrusions” category is simply a set of idiosyncratic intrusions from our data set. Consecutive intrusions were not considered to form a cluster.
