# Supplementary material for: A comparison of techniques for deriving clustering and switching scores from verbal fluency word lists
Source: Front Psychol. 2022 Sep 14;13:743557. doi: 10.3389/fpsyg.2022.743557 (PMC9518694; doi:10.3389/fpsyg.2022.743557)
Supplement: Supplementary file 2 [file Data_Sheet_1.pdf]

```
import numpy as np
import pickle
import operator

# This program demonstrates the functioning of the Needlman-Wunsch
algorithm
# for aligning two CMU pronunciations and calculating their
phonological
# similarity.

# The program depends on a measurement of similarity for each pair of
phonemes.
# Each phoneme is represented as a vector of phonetic features (e.g.,
continuant, voiced, labial, etc.)
# We calculated the euclidean distance between each pair of phonemes
using these vectors.
# The euclidean distances are stored in this pkl file:
with open("CMU_phoneme_euclideans_normed.pkl", "rb") as fh:
    euclideans = pickle.load(fh)

# Because the program depends on similarity and not distance, we
transform
# the euclidean distances to lie on the interval [-1, 1], with the
most
# similar pairs being rated 1 (i.e., identical phonemes) and the least
similar
# being rated -1 (e.g., 'F', 'AY')

# Transform distances to be between -1 and 1
euclideans = { k: (float(v) - 1) * -1 for (k, v) in
euclideans.items() }

# print(euclideans)
# print({ (k, v) for (k, v) in euclideans.items() if v ==
min(euclideans.values()) })

def strip_emphasis(p):
    return ''.join([ c for c in p if c not in
'()0123456789' ]).strip()

with open("animal_pronunciations.txt", "r") as fh:
    lines = [ el.split(':') for el in fh.readlines() ]
    tups = [ (el[0].strip(), [ strip_emphasis(x) for x in
el[1].strip().split('.') ]) for el in lines ]
    animal_pronuns = dict(tups)

with open("CMU_f-words.pkl", "rb") as fh:
    efs = { strip_emphasis(k): strip_emphasis(v).split('.') for (k, v)
in pickle.load(fh) }
```

```
pronuns = {**animal_pronuns, **efs}

def phonemic_edit_distance(s0, s1, dist=euclideans):
    m = len(s0)
    n = len(s1)
    mat = np.zeros((m+1, n+1))
    for i in range(m+1):
        mat[i, 0] = -1 * i
    for j in range(n+1):
        mat[0, j] = -1 * j
    for i in range(1, m+1):
        for j in range(1, n+1):
            upper = (-1 + mat[i-1, j])
            left = (-1 + mat[i, j-1])
            top_left = mat[i-1, j-1] + dist[(s0[i-1], s1[j-1])]
            mat[i, j] = max(upper, left, top_left)
    print(mat)
    return mat[m, n]

def pad(s):
    if len(s) == 1:
        return s + ' '
    else:
        return s

def poswa(s0, s1, dist=euclideans):
    # phonemic overlap score with alignment strings
    m = len(s0)
    n = len(s1)
    s0_align = []
    s1_align = []
    align_mat = np.zeros((m+1, n+1))
    mat = np.zeros((m+1, n+1))
    for i in range(m+1):
        mat[i, 0] = -1 * i
    for j in range(n+1):
        mat[0, j] = -1 * j
    for i in range(1, m+1):
        for j in range(1, n+1):
            upper = (-1 + mat[i-1, j])
            left = (-1 + mat[i, j-1])
            top_left = mat[i-1, j-1] + dist[(s0[i-1], s1[j-1])]
            directions = [upper, left, top_left]
            index, value = max(enumerate(directions),
key=operator.itemgetter(1))
            align_mat[i, j] = index
            mat[i, j] = value
    i = m
    j = n
    while i > 0 or j > 0:
```

```
        arrow = align_mat[i, j]
        if arrow == 0:
            s0_align = [pad(s0[i-1])] + s0_align
            s1_align = ['--'] + s1_align
            i -= 1
        elif arrow == 1:
            s0_align = ['--'] + s0_align
            s1_align = [pad(s1[j-1])] + s1_align
            j -= 1
        else:
            s0_align = [pad(s0[i-1])] + s0_align
            s1_align = [pad(s1[j-1])] + s1_align
            i -= 1
            j -= 1
    return (mat[m, n], '.'.join(s0_align), '.'.join(s1_align))

def pprint_pos(tup):
    (score, s1, s2) = tup
    print("__________________")
    print(f"score: {tup[0]}")
    print(' '.join(s1.split('.')))
    print(' '.join(s2.split('.')))
    print("------------------")

# The poswa function returns the best possible overlap between CMU
pronunciation strings
# as well as the phonological similarity score.
pprint_pos(poswa(pronuns['bandicoot'], pronuns['cougar']))
# Similarity score of -4.17

pprint_pos(poswa(pronuns['flies'], pronuns['fleas']))
# Similarity score of 3.04
```
