# Supplementary figures and images for: A comparison of techniques for deriving clustering and switching scores from verbal fluency word lists
Source: Front Psychol. 2022 Sep 14;13:743557. doi: 10.3389/fpsyg.2022.743557 (PMC9518694; doi:10.3389/fpsyg.2022.743557)

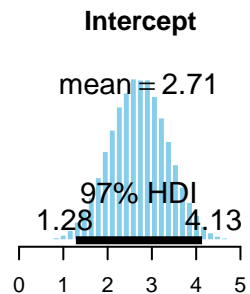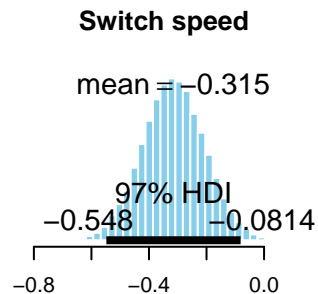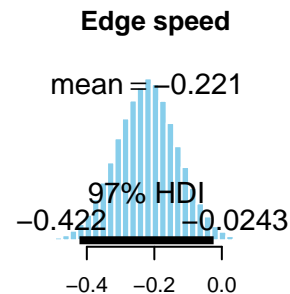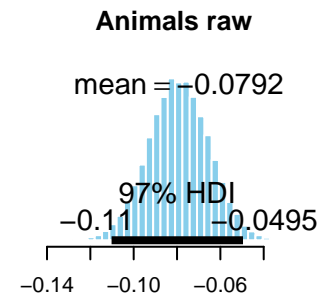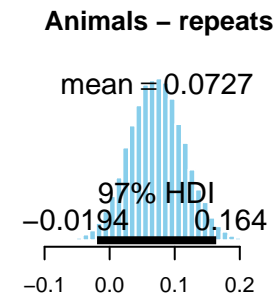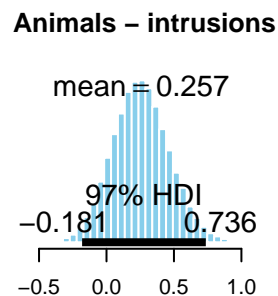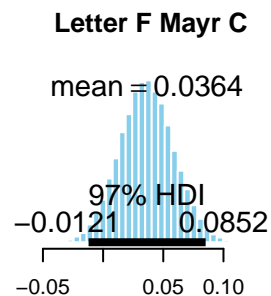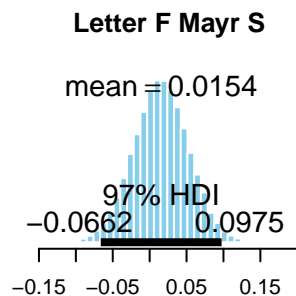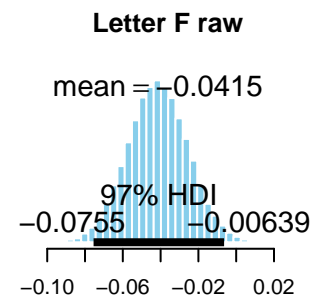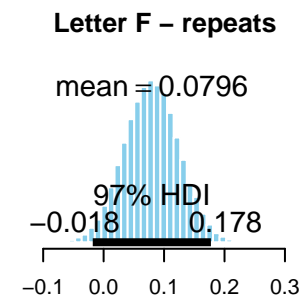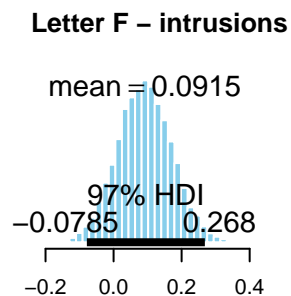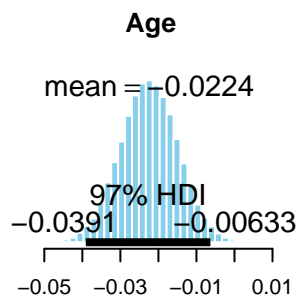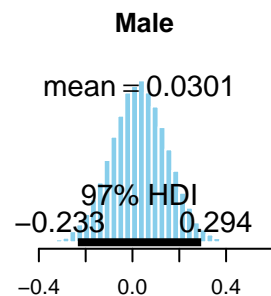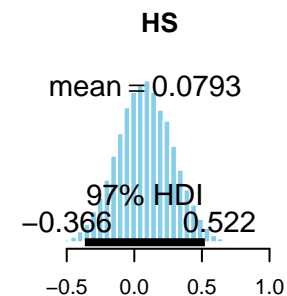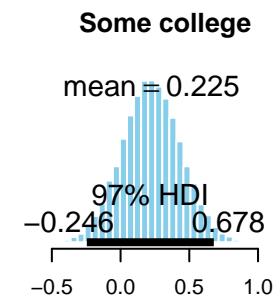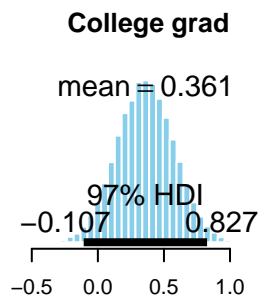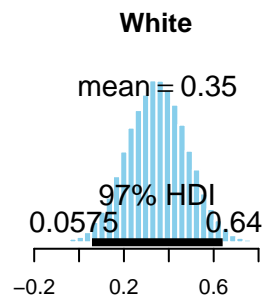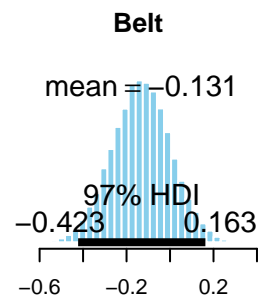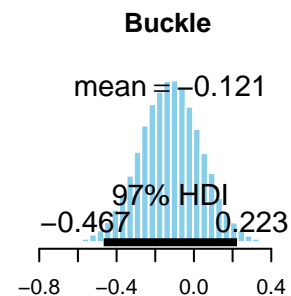

All ICI  
cases  
analysis

Supplement: Supplementary file 3 [file Image_1.pdf]

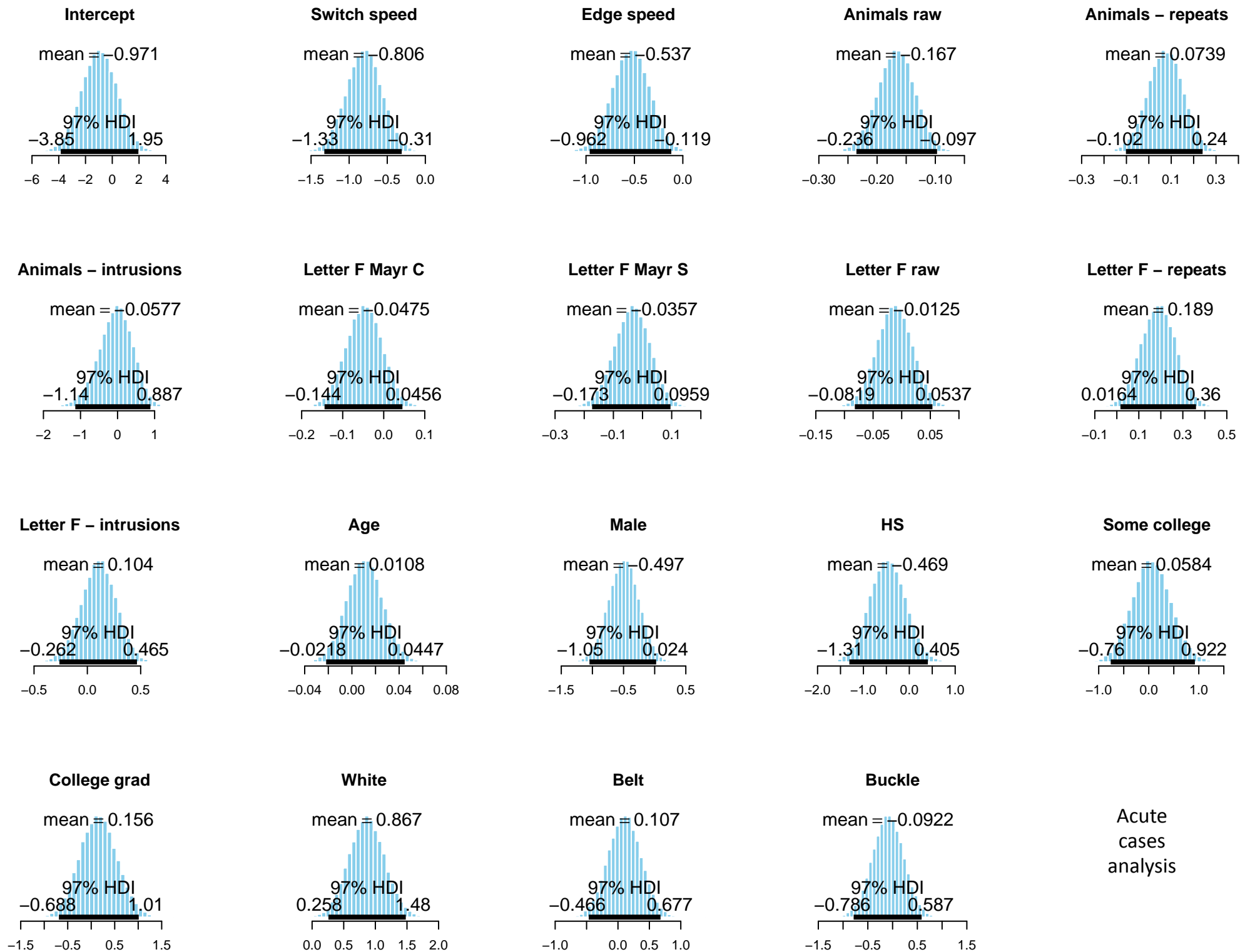

Supplement: Supplementary file 4 [file Image_2.pdf]

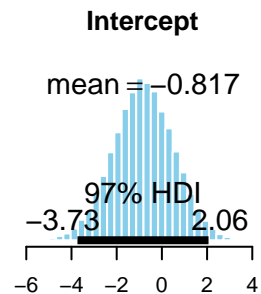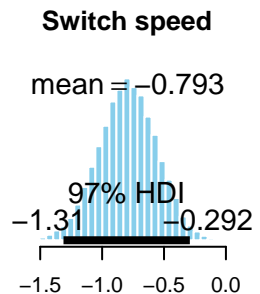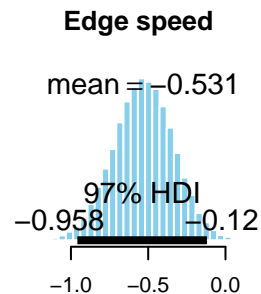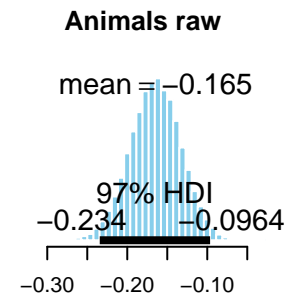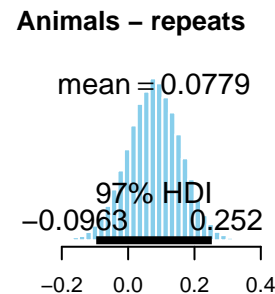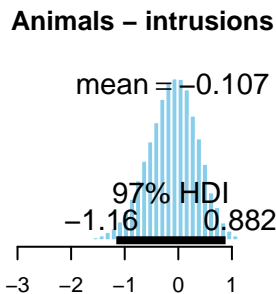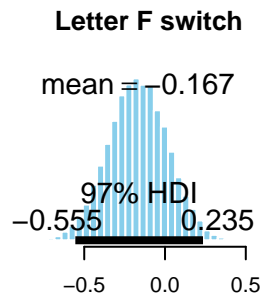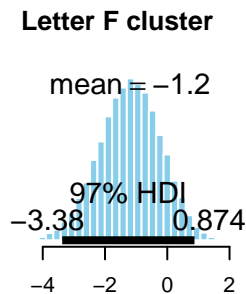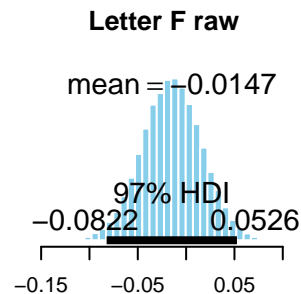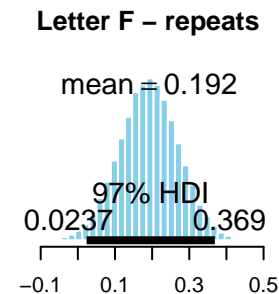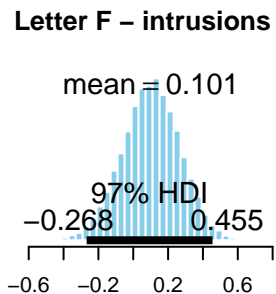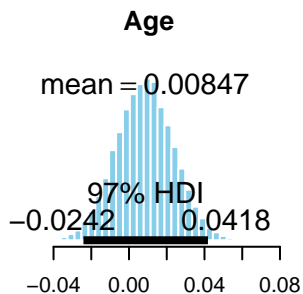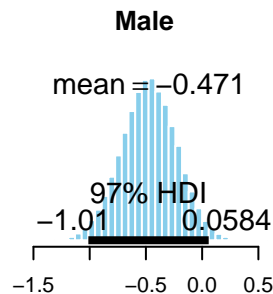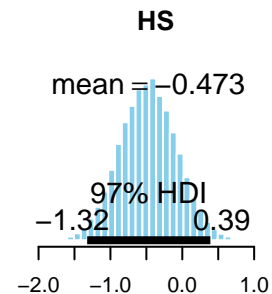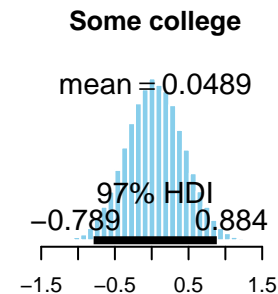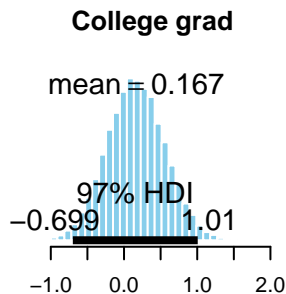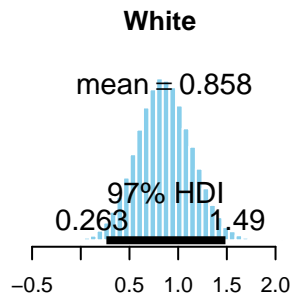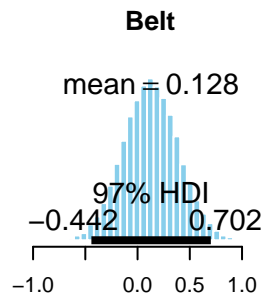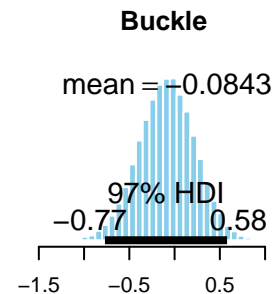

Progressive  
cases  
analysis

Supplement: Supplementary file 5 [file Image_3.pdf]
